# A modelling study of OH, NO_3_ and H_2_SO_4_ in 2007–2018 at SMEAR II, Finland: analysis of long-term trends†

**DOI:** 10.1039/d1ea00020a

**Published:** 2021-08-04

**Authors:** Dean Chen, Carlton Xavier, Petri Clusius, Tuomo Nieminen, Pontus Roldin, Ximeng Qi, Lukas Pichelstorfer, Markku Kulmala, Pekka Rantala, Juho Aalto, Nina Sarnela, Pasi Kolari, Petri Keronen, Matti P. Rissanen, Ditte Taipale, Benjamin Foreback, Metin Baykara, Putian Zhou, Michael Boy

**Affiliations:** Institute for Atmospheric and Earth System Research/Physics, University of Helsinki P.O. Box 64 00014 Helsinki Finland putian.zhou@helsinki.fi; Institute for Atmospheric and Earth System Research/Forest Sciences, University of Helsinki P.O. Box 64 00014 Helsinki Finland; Division of Nuclear Physics, Department of Physics, Lund University P.O. Box 118 SE-22100 Lund Sweden; Joint International Research Laboratory of Atmospheric and Earth System Sciences, School of Atmospheric Sciences, Nanjing University Nanjing 210023 China; Aerosol Physics Laboratory, Physics Unit, Faculty of Engineering and Natural Sciences, Tampere University Tampere Finland; Climate and Marine Sciences Department, Eurasia Institute of Earth Sciences, Istanbul Technical University Maslak 34469 Istanbul Turkey

## Abstract

Major atmospheric oxidants (OH, O_3_ and NO_3_) dominate the atmospheric oxidation capacity, while H_2_SO_4_ is considered as a main driver for new particle formation. Although numerous studies have investigated the long-term trend of ozone in Europe, the trends of OH, NO_3_ and H_2_SO_4_ at specific sites are to a large extent unknown. The one-dimensional model SOSAA has been applied in several studies at the SMEAR II station and has been validated by measurements in several projects. Here, we applied the SOSAA model for the years 2007–2018 to simulate the atmospheric chemical components, especially the atmospheric oxidants OH and NO_3_, as well as H_2_SO_4_ at SMEAR II. The simulations were evaluated with observations from several shorter and longer campaigns at SMEAR II. Our results show that daily OH increased by 2.39% per year and NO_3_ decreased by 3.41% per year, with different trends of these oxidants during day and night. On the contrary, daytime sulfuric acid concentrations decreased by 2.78% per year, which correlated with the observed decreasing concentration of newly formed particles in the size range of 3–25 nm with 1.4% per year at SMEAR II during the years 1997–2012. Additionally, we compared our simulated OH, NO_3_ and H_2_SO_4_ concentrations with proxies, which are commonly applied in case a limited number of parameters are measured and no detailed model simulations are available.

## Abstract 2

We applied the 1D model SOSAA to simulate the atmospheric concentrations of OH, NO_3_, and H_2_SO_4_ from 2007 to 2018 at SMEAR II. The results showed that daily OH increased by 2.39% per year and NO_3_ and H_2_SO_4_ decreased by 3.41% per year and 2.78% per year, respectively.

## Graphical abstract

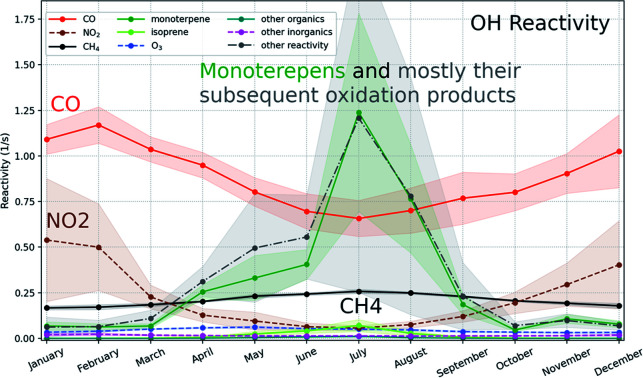

**Environmental significance**

The atmospheric oxidants (OH, O_3_ and NO_3_) are crucial in the oxidation processes of CH_4_, CO, SO_2_, *etc.*, and play a significant role in climate forcing and environmental processes. By using a process-based model with comprehensive datasets at a boreal forest measurement station, this study provides a long-term trend analysis of these atmospheric oxidants, which gives an insight into (1) how the atmospheric oxidation capacity evolves in a boreal forest background and (2) what are the local changes in the environment.

## 1. Introduction

Understanding the atmospheric oxidants (OH, O_3_ and NO_3_), their reactions and related processes is important as they are the main “cleaning protagonists” of the atmosphere. Many trace gases such as methane (CH_4_), volatile organic compounds (VOCs), nitrogen oxides (NO_*x*_ = NO + NO_2_) and sulfur dioxide (SO_2_) are removed from the atmosphere by oxidation reactions. During the day, the hydroxyl radical (OH) is the dominant oxidant produced by photochemical processes in the troposphere.^1^ Since there is no sunlight at night, the nighttime concentration of OH is significantly lower and other oxidants dominate: ozone (O_3_), during the daytime, is formed by OH radical reactions with VOCs in the presence of NO_*x*_, and the nitrate radical (NO_3_) is generated mainly by the reaction of NO_2_ with O_3_.^2–5^ In general, OH is considered to contribute the most to the atmospheric oxidation capacity,^6–10^ while O_3_ and NO_3_ play a minor but nonetheless significant role.^11^ Oxidation of VOCs by OH, O_3_ and NO_3_ affects air quality and climate, as well as regional and global budgets of reactive nitrogen, ozone, and secondary organic aerosols (SOAs).^12–15^

According to the latest global chemistry-transport model simulations, which used state-of-the-art new particle formation (NPF) parameterizations from the cloud chamber experiments in CERN,^16,17^ around 96% (ref. 18) or 100% (ref. 19) of the present-day NPF can be explained by H_2_SO_4_ clustering with ammonia, ions or organic compounds. Roldin *et al.* (2019)^15^ very recently observed NPF by considering sulfuric acid together with ammonia and/or ELVOCs during two periods in spring 2013 and 2014. Hence it is crucial to know the concentrations of H_2_SO_4_ for all NPF analyses and how they have changed in the past and will change in the future.

As biogenic sources dominate the global atmospheric VOC budget,^20,21^ it is important to understand the dynamics of biogenic emissions and their consequences to atmospheric processes. The boreal zone is the world's second largest forest region, after tropical forests,^22,23^ and boreal vegetation is dominated by evergreen coniferous trees that produce significant amounts of biogenic VOCs (BVOCs), mainly isoprene (C_5_H_8_), monoterpenes (C_10_H_16_) and sesquiterpenes (C_15_H_24_).^24–26^ Studies of the OH-reactivity in forest canopies have suggested large emissions of unknown reactive BVOCs.^9,27,28^

Studies on long-term trends of oxidants can provide an insight into how the atmospheric oxidation capacity evolves against the background of climate and local changes in the environment. Several studies have investigated the trends of atmospheric oxidants in Europe. In their studies, Wilson *et al.* (2012)^29^ and Yan *et al.* (2018)^30^ showed a general decreasing trend in ozone concentrations due to the decrease in NO_*x*_-emissions. Numerous studies have investigated global OH trends using chemical transport models or retrieval of remote sensing of methylchloroform (CH_3_CCl_3_, MCF).^31–33^ Prinn *et al.* (2001)^32^ predicted an overall global negative average OH trend of −0.64% per year between 1978 and 2000, whereas in a newer study, Montzka *et al.* (2011)^34^ found a small interannual OH variability, indicating that global OH is generally well buffered against perturbations. *In situ* long inter-annual OH measurements are relatively rare. However, one study by Rohrer *et al.* (2006)^35^ showed that there was no trend in the level of OH in the Hohenpeissenberg data set during the studied period 1999–2003 (estimated the annual trend to be less than ±2.5% per year) and that there was a positive correlation (*r* = 0.941) between OH and the photolysis frequency of ozone, J(O^1^D). Long term trends of NO_3_ are rarely studied, and only a few modelling studies on the long-term NO_3_ trend exist.^36^

A considerable number of field campaigns, in which OH concentrations were measured, have been compared to the results of modelling simulations.^37–39^ Most modelling studies reproduced the OH concentration within the uncertainty range of the OH measurements, including clean^40–45^ and urban areas.^46–51^ At the Station for Measuring Ecosystem – Atmosphere Relation (SMEAR II),^52^ located in Hyytiälä, Finland, OH concentrations were measured in two campaigns: European Integrated project on Aerosol, Cloud, Climate, and Air Quality Interactions (EUCAARI, 2007–2010)^53^ and Hyytiälä United Measurement of Photochemistry and Particles – Comprehensive Organic Particle and Environmental Chemistry (HUMPPA-COPEC).^54^ The results from these campaigns showed that modelled OH values were slightly overestimated compared to the measured values.^39,55^ During the HUMPPA-COPEC campaign in 2010 (ref. 54) and the IBAIRN (Influence of Biosphere – Atmosphere Interactions on the Reactive Nitrogen budget) campaign in 2016 (ref. 56), NO_3_ concentrations at SMEAR II were also measured, but most of the time the values were close to the limit of detection (LOD) of the instrument.^56^ However, due to the importance of the NO_3_ radical in the oxidation of BVOCs, Peräkylä *et al.* (2014)^57^ developed a NO_3_ proxy which was also used by Kontkanen *et al.* (2016)^58^ to derive the long-term trend of monoterpene concentrations at SMEAR II.

The aim of this study is to provide an insight into the long-term trend of the atmospheric oxidation capacity at the boreal forest in Finland from 2007 to 2018. Based on this, we estimate how the H_2_SO_4_ concentration has changed during this period, and how this could affect the frequency of new particle formation events at this site.

## 2. Methods

### 2.1. SMEAR II

The long-term measurements analyzed in this study were conducted at the SMEAR II station located in Hyytiälä (61°50′51′′N, 24°17′41′′E), Southern Finland.^59^ The station is surrounded by a 56 year old (in 2018) pine dominated forest that also contains Norway spruces and deciduous trees.^60^ SMEAR II is a unique field station with continuous measurements of physical, chemical and biological phenomena, processes and interactions between these elements. A detailed description of the site can be found on the SMEAR II website (https://www.atm.helsinki.fi/SMEAR/index.php/smear-ii).

One of the major changes that affects the general trend of atmospheric composition is the growing vegetation. The mean height of dominant trees within 200 m from the measurement tower was 16.9 m in the year 2007 and increased to 20.5 m in 2018. The overstory canopy depth was 8 m in the year 2007 and increased to 8.63 m by 2014. Since the year 2014, it remained constant. The all-sided LAI (Leaf Area Index) of all trees in July increased from 5.5 m^2^ m^−2^ in 2007 to 5.9 m^2^ m^−2^ in 2015. After 2015, it was considered the same as in 2015. The dry biomass of the foliage in the year 2007 was 0.51 kg m^−2^ and reached 0.58 kg m^−2^ in 2018.

In this study, selected measurements during the period 2007 to 2018 from the SMEAR II station were used as input for the model simulations; partly to nudge the meteorological parameters to the observations (temperature, absolute humidity, wind speed and direction) or as continuous input for selected gases (O_3_, NO_*x*_, SO_2_, CO and CH_4_), solar irradiance (global short wave radiation and photosynthetically active radiation), soil properties (soil temperature, soil water content and soil heat flux) and particle condensation sink (calculated from the particle size distributions which are measured by DMPS and APS). The input of O_3_, NO_*x*_, SO_2_, CO and CH_4_ can be considered to account for the effects of local and regional transport or large scale variation of these species. The anthropogenic VOCs were not included in this study, but the anthropogenic influence at SMEAR II is low with the nearest significant city (about 200 000 inhabitants) located 60 km southwest.^61^ A detailed description of the station instrumentation (parameter, location, time resolution, method, and temporal coverage) is available on the SMEAR II website under “List of the measurements” https://www.atm.helsinki.fi/SMEAR/index.php/smear-ii/measurements.

Although SMEAR II already started its operational work in the late 90's, we decided to focus our long-term modelling activities from the year 2007 onwards. The reason was the NO_*x*_ monitoring technique and the fact that the NO_*x*_ concentrations have a large impact on the simulated OH concentrations. Until February 2007, a molybdenum converter was used to convert NO_2_ to NO. However, this technique also measures other nitrogen compounds (*e.g.*, nitric acid, nitrous acid, and PAN) which are misinterpreted as NO and consequently the NO level is overestimated. Since March 2007, a photolytic blue light converter was used for only converting NO_2_ to NO, which enables more accurate data collection of NO (see Fig. S1 in the ESI†).

### 2.2. SOSAA

SOSAA (a model to simulate the concentrations of organic vapors, sulfuric Acid and aerosols) is a 1-D chemistry transport model used to study the atmospheric composition inside the planetary boundary layer. In the past, SOSAA has been applied to study the characteristics of oxidant reactivities,^9,27,28^ oxidation of trace gases,^55^ emission of BVOCs,^62^ and vertical exchange and dry deposition of ozone^63^ and BVOCs,^64^ respectively, as well as new particle formation and growth of sub-3 nm particles.^65^ SOSAA is written in Fortran and parallelized with MPI (Message Passing Interface). In this study, four different modules were used: (1) the meteorological module, which is derived from SCADIS;^66–68^ (2) the BVOC emission module, which is a modified version of MEGAN2.04 (Model of Emissions of Gases and Aerosols from Nature);^21^ (3) the chemistry module, which is created by KPP,^69^ with the chemical mechanism generated by MCM3.3.1 (see https://www.mcm.leeds.ac.uk/MCM);^70–72^ and (4) the gas dry deposition module, which is modified from MLC-CHEM.^63,64,73^ SOSAA describes the atmospheric boundary layer evolution and the vertical mixing of the chemical species in 51 vertical layers, from the surface up to 3 km. The simulation time step is 10 s for the meteorology module and 60 s for other modules.

The meteorological module includes the prognostic equations for the horizontal wind vector, air temperature and absolute humidity. In this study, these prognostic variables at the upper boundary of the model domain were constrained with the ERA-Interim reanalysis data which were provided by the European Centre for Medium-Range Weather Forecast (ECMWF).^74^ In the lower part of the model domain from 4.2 m to 125 m above the ground, the air temperature, wind vector and absolute humidity were nudged to the vertically interpolated measurement data at SMEAR II with a nudging factor of 0.05, which represents the force of regional transport. The incoming short-wave and photosynthetically active radiation (PAR) at the canopy top, as well as the soil properties (soil temperature, soil water content and soil heat flux) were directly taken as input from SMEAR II measurements. The short-wave radiation was provided by the measurement data at SMEAR II, and the radiative transfer module from the ADCHEM model^75^ was used to split the observed radiation into the direct, diffuse, downward and upward radiation components. The radiative transfer module used the quadrature two-stream approximation scheme developed by Toon *et al.* (1989).^76^ All of the meteorological input data mentioned above were linearly interpolated to 10 s time resolution to match the simulation time step.

The standard emission potentials of the emitted BVOCs at SMEAR II, which were used to calculate the emission rates, refer to the values suggested in Zhou *et al.* (2017b).^64^ The chemistry scheme was derived from the one used in Zhou *et al.* (2017b)^64^ but with a newer MCM version 3.3.1. For the reactions of the stabilized Criegee intermediates (sCIs), we diverted from the MCM and instead used newer obtained reaction rates. For the sCIs from α-pinene, β-pinene and limonene, we have used the rates from Mauldin III *et al.* (2012)^77^ similarly to “Scenario C” in Boy *et al.* (2013).^55^ For the sCIs from isoprene, we used the rates from Welz *et al.* (2012)^78^ as done in “Scenario D” in Boy *et al.* (2013).^55^

The measured mixing ratios of CO, O_3_, NO, NO_2_ and SO_2_ at the height levels 4.2, 8.4, 16.8, 33.6, 50.4, 67.2, 101 and 125 m were averaged and then used as the input values for all the layers in the model. The LODs of SO_2_, NO, O_3_ and NO_2_ were set to 0.06 ppb, 0.05 ppb, 0.3 ppb and 0.1 ppb, respectively (Dr Pasi Kolari, personal discussion). However, for all these four species there exist several long periods when the measured values were below the LOD. In order to prevent the model from noise interference, which has too low values, all the values that are below the LOD are set to the LOD. We also did test runs by setting all values below the LOD to LOD/2. However, the model results showed a stepwise increase in the simulated OH, NO_3_ and H_2_SO_4_ concentrations at all times when the input data went from the LOD to LOD/2. So, we decided to use the LOD as a threshold in case the values are below the LOD for the four gaseous compounds discussed above. There are several other methods used in the literature to overcome this problem such as the “Uniform Fill-In” or the “Log Fill-In” methods discussed and tested by Cohen and Ryan (1998).^79^ However, as all the data below the LOD are unknown, no method predicts their distribution correctly which makes it difficult to choose a single technique that will be best at all times for various parameters. In Table S1 in the ESI,† we calculated the amount of data points for SO_2_ and NO above the LOD (the two parameters with the highest amount of data below the LOD) for different percentile ranges for each year to investigate if a trend in the below LOD data exists.

The measured CH_4_ concentrations in 2014 were used as input in SOSAA for the year 2014. For other years, an annual global growth rate of 6 ppb per year was assumed, and the input time series of CH_4_ concentrations were thus added (after 2014) or subtracted (before 2014) a multiple times of 6 ppb from the time series in 2014 according to the year difference. The growth rate was chosen from the ‘NASA Earth Observatory’ website and represents the methane increase in 2007–2013 (https://www.earthobservatory.nasa.gov/images/87681/a-global-view-of-methane).

The condensation sinks (CS) for H_2_SO_4_ and HNO_3_ were provided as an input for the model. The CS was calculated based on the particle size distribution measured by using a DMPS (particles with diameters of 3–1000 nm) and an APS (particles with aerodynamic diameters of 0.5–20 μm) system,^52,80^ and the hygroscopic growth effect was corrected based on Laakso *et al.* (2001).^81^ Similarly, for the meteorological input data, the input mixing ratios and the CS were also linearly interpolated to 60 s time resolution to match the simulation time step of the emission and chemistry modules.

### 2.3. Statistical methods

The daily/daytime/nighttime trends of variables were calculated based on their daily/daytime/nighttime mean or median values. Whether to use mean or median for a variable is determined by its data value distribution. If the data are logarithmically distributed (O_3_, CO, CS, EM-MON, MON, OH, HO_2_, H_2_SO_4_, NO_2_, N_2_O_5_, and NO_3_), the median values are used. Here we should notice that although the data value distributions of SO_2_ and NO are also logarithmically distributed, we still used their mean values. The reason is that more than 50% of their measured concentrations lie below the LOD, which results in that their median values are equal to the LOD. For other variables (temperature, RH, and solar irradiance), the mean values were used. For the logarithmically distributed variables (besides the variables mentioned above, SO_2_ and NO are also included here), the daily/daytime/nighttime linear trend fittings were conducted on the logarithm with base 10 of their respective median or mean values. For other variables (temperature, RH, and solar irradiance), the linear trend fittings were performed directly on their respective mean values.

Bootstrapping was used to estimate the confidence interval of the trend.^82,83^ We first fitted a linear trend to the time series and created a new data set by taking random samples from the original residuals (differences of the data values and the fitted linear trend) and adding these to the linear part. Then a new linear fit was made to this new data set. This procedure was repeated several times (typically 10 000 iterations). Here the idea was to test the monotonicity of the trend. The smaller the differences in the fitted trends were after many such iterations, more likely was the original trend to be monotonic. To obtain the confidence interval, we examined the 5^th^ to 95^th^ percentile range of the slopes obtained from bootstrapping iterations: if all of the slopes in this range were either positive or negative (thus not containing a zero trend), we concluded that the likelihood of the presence of a trend was higher than 95% (*p* < 0.05) and thus statistically significant.

To get another estimate of the monotonicity of the trends, we also used the Mann–Kendall test for autocorrelated seasonal data,^84,85^ and the *p*-values are reported in Table 1 under *P*_MK_. The MK test is more conservative, but both our tests agree in the sense that a wider confidence interval or larger *P*_MK_ value indicates larger yearly variation, and hence the prognostic capacity of the trend is smaller.

**Table 1:** Yearly trends of individual parameters calculated by two different statistical methods (RLM = robust linear method, MK = Mann Kendall) and their seasonal trends calculated by the RLM. The numbers in brackets are the 90% confidence intervals (first and second numbers show the 5th and 95th percentiles of the trend slopes obtained from 10 000 bootstrapping iterations, respectively) for the RLM values, and *P*_MK_ values of MK numbers. The first, second and third rows for each parameter represent daily, daytime and nighttime values, respectively. Whether the median or mean values of different parameters were used to calculate the trends are also shown in the bracket after the parameter names. Except the emissions of the monoterpenes, which were averaged over the forest canopy height (18 m), all other parameters use the median or mean values from the height level of 20–40 m. The significant trends are marked in bold font. Detailed statistical methods are explained in Section 2.3

Parameter	Time	Yearly trend		Seasonal trend			
		RLM	MK	Winter	Spring	Summer	Autumn
Monoterpene emission rate (median)	Daily	+0.80 (−0.06, +1.90)	+0.72 (0.152)	+2.07 (−0.98, +5.87)	**+0.53 (+0.06, +1.96)**	+1.00 (−0.23, +2.30)	+0.40 (−1.04, +1.27)
	Day	+0.73 (−0.14, +1.82)	+0.69 (0.166)	+1.88 (−1.30, +5.92)	**+0.74 (+0.31, +2.13)**	+0.80 (−0.41, +2.14)	+0.27 (−1.23, +1.16)
	Night	+0.76 (−0.17, +1.98)	+0.67 (0.230)	+2.06 (−0.83, +5.90)	+0.26 (−0.42, +1.62)	**+1.14 (+0.05, +2.44)**	+0.38 (−1.02, +1.39)
Monoterpene concentration (median)	Daily	**+3.43 (+1.95, +5.27)**	**+3.11 (0.010)**	**+4.24 (+2.00, +7.12)**	**+2.87 (+1.39, +5.22)**	**+3.06 (+0.32, +6.12)**	**+3.66 (+2.38, +4.56)**
	Day	**+3.15 (+1.75, +4.98)**	**+2.82** (**0.014)**	**+3.89 (+1.58, +7.03)**	**+2.78 (+1.18, +5.28)**	**+2.65 (+0.24, +5.40)**	**+3.36 (+2.23, +4.18)**
	Night	**+3.60 (+2.08, +5.43)**	**+3.46** (**0.009)**	**+4.09 (+1.86, +6.99)**	**+2.74 (+1.36, +4.95)**	**+4.11 (+0.75, +7.76)**	**+3.71 (+2.32, +4.71)**
OH (median)	Daily	**+2.39 (+0.95, +3.33)**	**+1.84** (**0.036)**	**+9.65 (+3.67, +16.02)**	**+1.37 (+0.73, +2.93)**	−0.85 (−2.24, +0.25)	**+3.47 (+1.49, +4.83)**
	Day	+0.91 (−0.81, +2.10)	+1.10 (0.236)	**+6.87 (+1.21, +11.41)**	+0.68 (−0.46, +2.11)	−1.11 (−2.41, +0.04)	+1.14 (−1.04, +2.46)
	Night	**+3.31 (+2.01, +4.62)**	**+2.40** (**0.022)**	**+10.25 (+3.95, +17.32)**	**+2.25 (+1.29, +3.74)**	+0.26 (−0.80, +1.32)	**+4.54 (+2.39, +6.29)**
HO_2_ (median)	Daily	**+3.34 (+2.00, +4.38)**	**+2.64 (0.013)**	**+10.75 (+3.97, +18.03)**	**+1.08 (+0.05, +3.03)**	+0.78 (−0.68, +2.02)	**+4.52 (+2.36, +5.97)**
	Day	**+2.10 (+0.94, +3.10)**	**+1.48** (**0.037)**	**+12.15 (+3.94, +21.27)**	−0.05 (−1.26, +1.67)	+0.11 (−1.27, +1.43)	**+2.33 (+0.26, +3.49)**
	Night	**+3.96 (+2.57, +5.32)**	**+3.19 (0.015)**	**+10.26 (+3.72, +17.74)**	**+2.10 (+0.95, +3.91)**	+1.49 (−0.03, +3.04)	**+5.39 (+3.05, +7.26)**
H_2_SO_4_ (median)	Daily	−1.54 (−4.60, +0.36)	−1.10 (0.521)	+2.96 (−1.64, +6.84)	−0.76 (−3.93, +2.84)	**−5.02 (−9.06, −1.12)**	+0.70 (−1.95, +3.06)
	Day	**−2.78 (−6.05, −0.63)**	−2.62 (0.183)	−0.54 (−5.69, +2.85)	−0.66 (−4.32, +2.89)	**−5.40 (−9.52, −1.07)**	−1.12 (−3.76, +0.95)
	Night	−0.58 (−3.28, +1.49)	−0.34 (0.829)	+3.19 (−1.00, +7.11)	−0.77 (−4.39, +2.97)	−3.48 (−6.89, +0.20)	+1.87 (−0.92, +4.54)
NO_3_ (median)	Daily	**−3.41 (−4.98, −2.09)**	**−3.40 (0.014)**	**−2.99 (−4.50, −1.47)**	**−3.00 (−5.96, −0.26)**	**−3.38 (−5.54, −1.23)**	**−4.53 (−5.99, −2.73)**
	Day	**−2.43 (−3.90, −0.88)**	**−2.54 (0.033)**	−1.03 (−3.43, +2.24)	**−2.58 (−5.20, −0.12)**	**−2.89 (−5.25, −0.64)**	**−3.65 (−4.74, −2.24)**
	Night	**−4.22 (−5.90, −2.86)**	**−4.11** (**0.009)**	**−3.23 (−4.81, −1.90)**	**−3.80 (−6.55, −1.28)**	**−5.07 (−7.54, −2.68)**	**−5.27 (−6.92, −3.38)**
N_2_O_5_ (median)	Daily	**−8.24 (−12.18, −5.96)**	**−7.84 (0.013)**	**−12.61 (−18.96, −7.00)**	**−5.36 (−11.06, −0.90)**	−4.11 (−9.70, +1.76)	**−9.09 (−12.05, −4.71)**
	Day	**−6.90 (−10.39, −4.68)**	**−6.64** (**0.018)**	**−10.62 (−15.71, −5.63)**	−4.22 (−9.95, +0.26)	−2.52 (−8.36, +3.41)	**−8.28 (−10.88, −4.16)**
	Night	**−9.16 (−13.18, −6.93)**	**−8.68** (**0.010)**	**−12.69 (−19.48, −6.92)**	**−6.55 (−12.06, −2.14)**	**−6.68 (−12.16, −1.15)**	**−10.16 (−13.60, −5.46)**
SO_2_ (mean)	Daily	**−5.43 (−8.10, −3.65)**	**−3.91** (**0.013)**	**−10.18 (−17.00, −4.85)**	−4.10 (−7.73, −0.53)	**−4.57 (−7.75, −1.18)**	**−3.59 (−5.18, −1.98)**
	Day	**−5.00 (−7.67, −3.22)**	**−2.65** (**0.019)**	**−10.01 (−17.07, −4.08)**	−3.20 (−6.81, +0.26)	**−4.48 (−7.88, −0.88)**	**−2.76 (−4.03, −1.47)**
	Night	**−5.35 (−8.07, −3.55)**	**−3.07** (**0.013)**	**−9.97 (−16.71, −4.73)**	−4.54 (−8.29, −0.86)	**−3.91 (−7.13, −0.59)**	**−3.54 (−5.04, −1.94)**
O_3_ (median)	Daily	−0.11 (−0.86, +0.05)	+0.16 (0.612)	+0.74 (−0.49, +1.45)	+0.13 (−0.51, +0.82)	−0.28 (−1.12, +0.48)	+0.04 (−0.93, +0.98)
	Day	**−0.18 (−0.88, −0.01)**	+0.03 (0.884)	+0.89 (−0.31, +1.51)	+0.09 (−0.52, +0.75)	−0.37 (−1.16, +0.35)	−0.27 (−1.18, +0.60)
	Night	+0.04 (−0.76, +0.21)	+0.30 (0.330)	+0.67 (−0.59, +1.38)	+0.24 (−0.45, +0.95)	−0.02 (−0.89, +0.79)	+0.24 (−0.75, +1.27)
CO (median)	Daily	−0.46 (−1.62, +0.38)	−0.42 (0.473)	**−0.89 (−1.96, −0.28)**	**−0.96 (−1.72, −0.32)**	−0.54 (−2.50, +1.41)	+0.75 (−0.89, +2.44)
	Day	−0.47 (−1.63, +0.34)	−0.44 (0.456)	**−0.98 (−2.01, −0.39)**	**−0.96 (−1.71, −0.31)**	−0.54 (−2.47, +1.43)	+0.79 (−0.81, +2.45)
	Night	−0.43 (−1.63, +0.42)	−0.41 (0.499)	**−0.85 (−1.93, −0.24)**	**−0.97 (−1.74, −0.30)**	−0.53 (−2.41, +1.45)	+0.78 (−0.79, +2.48)
NO (mean)	Daily	−0.37 (−1.13, +0.08)	−0.02 (0.907)	**−2.61 (−4.96, −1.67)**	+0.63 (−0.84, +1.95)	−0.02 (−0.50, +0.49)	−0.27 (−0.80, +0.20)
	Day	**−0.91 (−2.12, −0.19)**	−0.13 (0.631)	**−5.93 (−10.15, −3.86)**	+0.80 (−1.18, +2.56)	−0.00 (−0.61, +0.68)	−0.65 (−1.61, +0.31)
	Night	+0.00 (−0.02, +0.02)	+0.00 **(**0.980)	−0.22 (−0.55, +0.10)	+0.01 (−0.01, +0.03)	−0.00 (−0.01, +0.00)	+0.02 (−0.03, +0.06)
NO_2_ (median)	Daily	**−3.80 (−5.99, −1.87)**	**−3.89 (0.031)**	**−8.49 (−12.49, −4.87)**	−2.32 (−6.07, +0.99)	−1.34 (−4.36, +1.92)	**−4.13 (−5.86, −1.96)**
	Day	**−3.33 (−5.52, −1.43)**	**−3.31 (0.045)**	**−8.61 (−12.52, −5.05)**	−1.48 (−5.21, +1.99)	−0.71 (−3.66, +2.45)	**−3.67 (−5.28, −1.53)**
	Night	**−4.18 (−6.26, −2.32)**	**−4.21** (**0.019)**	**−8.21 (−12.46, −4.53)**	**−3.08 (−6.18, −0.22)**	−2.00 (−4.95, +1.04)	**−4.64 (−6.66, −2.25)**
Global short wave radiation (mean, W m^2^ per year)	Daily	+0.24 (−0.85, +0.68)	+0.27 (0.110)	**−0.20 (−0.68, −0.22)**	+1.37 (−0.02, +3.43)	+0.85 (−1.25, +2.61)	+0.02 (−0.64, +0.30)
	Day	+0.26 (−1.54, +1.05)	+0.47 (0.177)	**−0.74 (−2.05, −0.80)**	+2.42 (−0.01, +5.52)	+0.90 (−1.74, +3.34)	−0.06 (−1.40, +0.63)
	Night	+0.01 (−0.00, +0.03)	+0.01 (0.353)	**+0.03 (+0.00, +0.07)**	−0.01 (−0.02, +0.00)	−0.00 (−0.06, +0.05)	−0.00 (−0.01, +0.01)
Temperature (mean, K per year)	Daily	**+0.08 (+0.02, +0.24)**	+0.03 (0.632)	+0.23 (−0.04, +0.68)	−0.02 (−0.10, +0.13)	−0.02 (−0.17, +0.14)	−0.03 (−0.14, +0.14)
	Day	**+0.08 (+0.02, +0.24)**	+0.03 (0.569)	+0.23 (−0.06, +0.67)	−0.02 (−0.10, +0.13)	−0.02 (−0.18, +0.14)	−0.03 (−0.14, +0.13)
	Night	**+0.08 (+0.03, +0.23)**	+0.02 (0.714)	+0.24 (−0.04, +0.69)	−0.03 (−0.11, +0.12)	−0.00 (−0.14, +0.14)	−0.02 (−0.14, +0.14)
Absolute humidity (mean, g m^3^ per year)	Daily	−0.11 (−0.47, +0.70)	−0.27 (0.343)	+0.21 (−1.23, +2.86)	−0.19 (−0.64, +0.51)	−0.26 (−0.67, +0.18)	−0.37 (−1.02, +0.11)
	Day	−0.07 (−0.44, +0.76)	−0.24 (0.417)	+0.20 (−1.22, +2.88)	−0.13 (−0.58, +0.59)	−0.26 (−0.69, +0.19)	−0.31 (−1.04, +0.11)
	Night	−0.17 (−0.55, +0.65)	−0.33 (0.287)	+0.15 (−1.30, +2.73)	−0.29 (−0.76, +0.40)	−0.29 (−0.69, +0.14)	−0.36 (−0.98, +0.10)
Condensation sink (median)	Daily	**−1.59 (−3.49, −0.12)**	−1.08 (0.356)	**−4.76 (−7.84, −2.95)**	−1.11 (−3.51, +1.51)	−0.04 (−1.64, +1.52)	−0.87 (−3.06, +1.07)
	Day	**−1.65 (−3.59, −0.20)**	−1.10 (0.334)	**−5.19 (−8.59, −3.36)**	−1.22 (−3.73, +1.51)	−0.13 (−1.73, +1.48)	−0.68 (−3.01, +1.45)
	Night	**−1.39 (−3.22, −0.02)**	−0.89 (0.419)	**−4.34 (−7.41, −2.64)**	−1.14 (−3.42, +1.36)	+0.25 (−1.32, +1.85)	−0.81 (−2.98, +1.14)

Finally, the relative changes (and the 90% confidence interval from the bootstrapping test) which are shown in Table 1 are in the linear scale for all variables, describing the actual change in % per year. The trend lines shown in time series plots are obtained with a 1 year running median (window size of ±182 days); see Ma *et al.* (2016)^86^ for a detailed description.

### 2.4. Uncertainties

The uncertainties in this study are mainly related to the uncertainties of the input variables or the use of parametrizations inside the model. Concerning the input data, the aerosol condensation sink, which predicts in our study how rapidly sulfuric acid and nitric acid will condense on pre-existing aerosols, has the highest uncertainty. In these calculations, we applied measured particle size number concentrations from SMEAR II. However, the uncertainty of the predicted CS values due to potentially different hygroscopic growth behaviour depending on the chemical composition of the particles is difficult to estimate and could have an effect on the simulated acid concentrations.

The second source of uncertainties is related to the meteorology module. It will be validated by comparison with measurements in the following section. The third uncertainty source is the large uncertainties of reaction rate coefficients in the applied chemistry schemes, and when the same reaction is studied by different groups with different techniques, the reaction rate coefficient may differ by a factor of 2 or even more.^87^ Moreover, for many reactions between OH and VOCs, no experimental data exist, so the reaction rate coefficients are only estimates, which increase the uncertainty even further.

The fourth uncertainty source is related to the emission module MEGAN. Guenther *et al.* (2012)^88^ estimated that the uncertainty associated with the annual global emissions of monoterpenes is a factor of three, and of methanol, acetone and acetaldehyde is a factor of two. However, in our simulations MEGAN was constrained by *in situ* measurement data, including the relevant meteorological variables and the standard emission factors, which decreased the uncertainty. Moreover, the uncertainty of the emission module can also be evaluated by comparing the measured and simulated monoterpene concentrations and fluxes in Section 3.3.

## 3. Results

The results will be presented in 6 sections: (1) validation of the meteorological module, (2) a short discussion of the trends of the measured parameters which are crucial for our model simulations, (3) BVOC model-measurement intercomparison and trends, (4) trends and campaign model-observation inter-comparisons of the hydroxyl and nitrate radicals, (5) trends and campaign model-observation inter-comparisons of sulfuric acid and (6) comparisons of proxies of OH, NO_3_ and H_2_SO_4_ with the model.

Table 1 shows the trends calculated by the method described in Section 2.3 of all relevant model output parameters for the whole day (daily), daytime and nighttime, respectively, and also shows the seasonal trends calculated with the RLM method and the 90% confidence interval for the same parameters. Here the daytime is defined as the time period between sunrise and sunset; nighttime is defined between sunset and sunrise and the daily values are averaged for 24 hours. In the following sections we will discuss the values of single parameters in more detail.

### 3.1. Meteorological data analyses

Meteorology is one of the major drivers for the change in atmospheric composition. We compared several measured meteorological parameters with the model outcome to validate the performance of the meteorological module in SOSAA. While temperature, water vapor and wind speed were nudged with the measurements, the heat fluxes and net radiation were simulated and their comparison with measurements provides an insight into the simulated energy balance above the forest canopy.

**Fig. 1:**
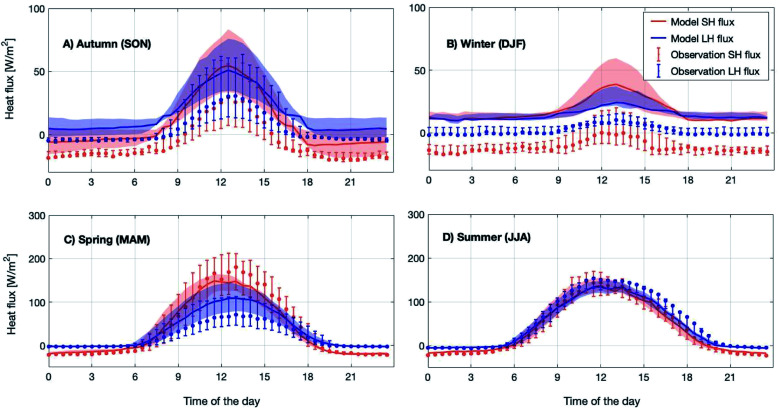
Measured (dots) and modelled (solid lines) diurnal median sensible (SH, red) and latent (LH, blue) heat fluxes above the SMEAR II station (23 m) averaged for four seasons over the period 2007–2018 in subplots (A–D). The letters in brackets in the subplot titles represent the months used for the individual seasons. The 25^th^–75^th^ percentile is shown as shades and vertical bars for modelled and measured fluxes, respectively.

Fig. 1 shows the modelled 12 year median diurnal cycles of sensible and latent heat fluxes for the four different seasons in 2007–2018. The comparison shows that the modelled sensible heat flux is within 25^th^–75^th^ percentiles of the measured ones throughout the whole diurnal cycle in spring and summer and during daytime in autumn. However in winter and during autumn nighttime, the model always overestimated the measured sensible heat flux.

The modelled latent heat flux during spring daytime is about 20 W m^−2^ lower compared to the observations, which could be related to the melting of the snow cover on the ground. Note that the snow cover is not explicitly modelled in SOSAA. However, during summer, the model and measurements show good agreement. For the winter and autumn months, the simulated latent heat flux shows a similar overestimation to the sensible heat flux. For other seasons, they are most of the time within the 25^th^–75^th^ percentile range. We want to point out that the measured fluxes in the winter and autumn months are very low (<10 W m^−2^) and an overall underestimation of heat fluxes during these periods is normal when applying the eddy covariance technique^89^ such as at SMEAR II; hence it is difficult to draw a conclusion on the accuracy of either the model or measurements during these periods.

**Fig. 2:**
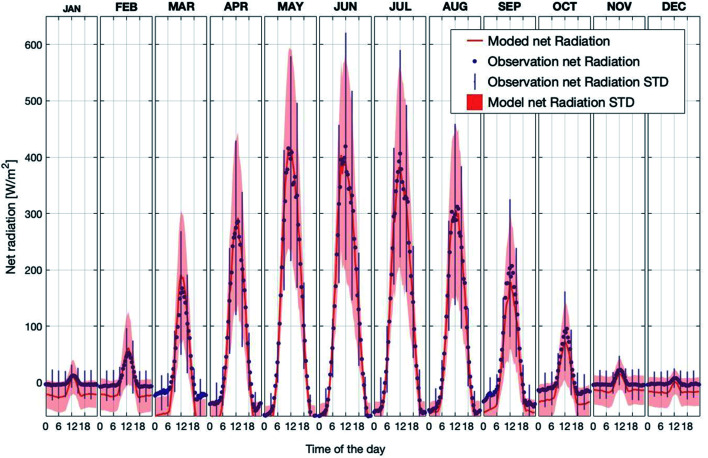
Observed and simulated average diurnal cycles of net radiation at SMEAR II for each month separately for the years 2007–2018 (STD = standard deviation).

Fig. 2 shows the observed and simulated average diurnal cycle of net radiation at 125 m for each month in the period 2007–2018. Here, the net radiation is calculated as the total incoming short- and long-wave radiation minus the total outgoing short- and long-wave radiation at the canopy top. Thus, positive values represent more incoming than outgoing radiation and *vice versa*. The modelled daytime values agree well with the measurements for all months. By contrast, the modelled nighttime net radiation underestimated the measurements by about ∼10 W m^−2^ to ∼50 W m^−2^ from September to December and from January to March. In general, the model is consistent with the measurements, and is able to capture the diurnal pattern and seasonal trend of net radiation above the canopy. Therefore, considering the simulation results of SH and LH discussed above, the model can predict a reasonable energy balance inside and above the canopy.

### 3.2. Trend of measured parameters

In Table 1 and Fig. S2,† the yearly and seasonal trends of selected measured parameters at SMEAR II are provided. Short wave global radiation, temperature, absolute humidity and condensation sink show no significant yearly trends. Only during the winter seasons, a clear daily decrease in the condensation sink by −3.96 (−7.48, −1.20) % per year is visible. This may partly be related to the reduction of primary aerosol emissions from traffic, industry and heating as pointed out by Nieminen *et al.* (2014).^90^

The main inorganic gases (CO, O_3_, NO, NO_2_ and SO_2_) that serve as input to SOSAA reflect the influence of human impact on a regional scale. Carbon monoxide, for example, has a lifetime of approximately 1–3 months,^91^ and reveals the impact of large regional to hemispherical features. Nitrogen oxides and sulfur dioxide have lifetimes of days to weeks, respectively, and they are mainly related to local or regional changes. At a rural station such as SMEAR II, their concentrations are often below the LOD of the instruments. The 12-year concentrations of these five measured trace gases all show a negative trend reflecting the decreased anthropogenic impact on these gases in Europe during the last few decades.^92^ This trend in Europe was also confirmed by the latest EAA report (No 12/2018). Our trend analyses of daily values show that CO, NO and O_3_ concentrations only depict a marginal not significant decrease, while the concentrations of SO_2_ and NO_2_ drop by −5.43 (−8.10, +3.65) % per year and −3.80 (−5.99, −1.87) % per year, concerning the whole day, respectively.

However, as pointed out in Section 2.3, more than half of the SO_2_ and NO measurements are below the LOD of the instruments. Table S1 (in the ESI†) shows that a fraction of the NO and SO_2_ measurements is below the LOD in a year-wise fashion. The time period with SO_2_ below LOD within one year has increased from 2007 to 2018, which points to an even stronger decrease of SO_2_ concentrations than the 5.43% per year mentioned above. For NO, the amount of data measured below the LOD are much higher but without any visible trend. Note that in the evolution of the mean values and the 90^th^ and 75^th^ percentiles for NO, no trend is observed concerning the number of days with NO concentrations below the LOD (see also the discussion in Section 2.3).

### 3.3. BVOC's comparisons and trends

#### 3.3.1. Validation of monoterpene model results

At SMEAR II, monoterpenes are the dominant BVOCs,^60^ and during summer and spring they are the major contributors to the OH reactivity of the measured organic compounds.^27,28^ Thus, accurate modelling of monoterpenes is a crucial component for calculating the OH concentration. Furthermore, the monoterpenes (at SMEAR II) are climatically important because they can be oxidized to form low volatile organic compounds (LVOCs) and hence contribute to secondary aerosol formation.^15^ Anthropogenic volatile organic compounds (AVOCs) are not included in this study but their concentrations at SMEAR II are small compared to those of BVOCs.^61^

**Fig. 3:**
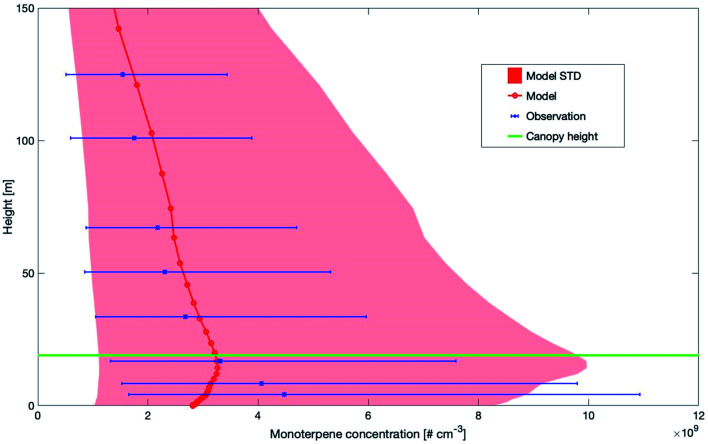
Vertical profiles of measured and modelled monoterpene median concentrations and ±1 standard deviation (STD) at SMEAR II, Finland for the years 2007–2014 and 2017–2018 (2015–2016 measurement data were not available). The canopy height is marked by a horizontal green line.

Fig. 3 shows the modelled *versus* the measured median monoterpene concentrations between 0 m and 150 m at SMEAR II. Both, the measurements and the simulations of monoterpene concentrations show an increase above the canopy. However, the peak from the measurements is at 4.2 m, while the model shows the highest values at 16.8 m. Modelled and measured values decrease at a similar rate above the canopy. The different height levels of the maximum could be related to the missing emission sources from understory vegetation and soil.^93,94^ Another reason for the decrease of the modelled concentrations inside the canopy could be related to the emission of monoterpenes from ground vegetation and soil as reported by Aaltonen *et al.* (2011).^95^ Currently, these sources of terpenes are not included in SOSAA and may explain the discrepancy in the lower canopy. For this reason, we compared the measured and modelled monoterpene concentrations for heights above the canopy.

**Fig. 4:**
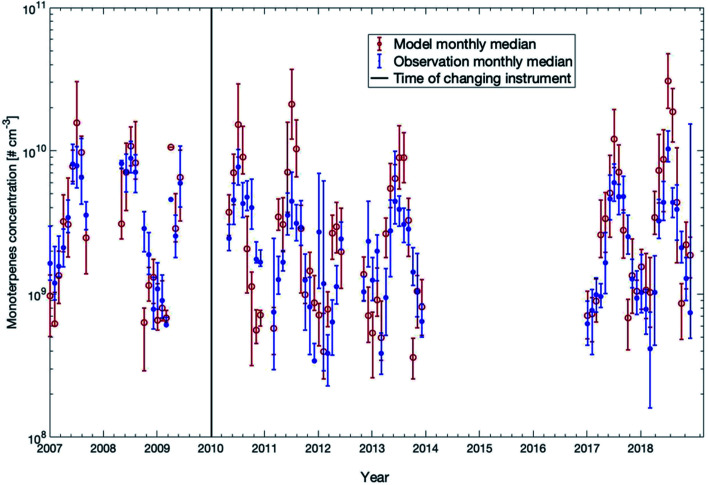
Measured and modelled monthly median values of monoterpene concentrations from 2007 to 2018 for the height interval 32–125 m. The measurement median data were obtained from the mean values of five measurement heights: 32 m, 54 m, 74 m, 101 m, and 125 m. The model median data were calculated from the mean of all the levels between 32 m and 125 m. The 25^th^ and 75^th^ percentiles for both data sets are shown as vertical bars.

In Fig. 4, we compared the measured monoterpene concentrations against the model outcome between 32 m and 125 m for all years except years 2014–2016 (measured data were not available for this period). The model slightly underestimated the monoterpene concentrations in winter while overestimated the values in summer. In years when the summer was exceptionally warm (*e.g.*, 2018), the model overestimated the monoterpene concentration by a factor of 2–3. The reason for this overshooting of the model during hot summers could be that the decrease of monoterpene emissions in the forest during drought is not accurately represented in the emission module MEGAN. In general, for more than 70% of the time, the modelled concentrations are within the range of 25^th^ and 75^th^ percentiles of the measured data points.

In order to compare the modelled and measured trends of monoterpene concentrations, we only consider the model data points when measurements were available to calculate the linear fittings or the trends. First, considering the measurement gap during 2014 to 2016, we can separate the datasets into two periods before and after the gap. One is from 2007 to 2013 and the other is from 2017 to 2018. The linear fittings show that the modelled trends of these two periods are −0.72 × 10^8^ molecules per cm^3^ per year and 19 × 10^8^ molecules per cm^3^ per year, and the measured trends are −6.8 × 10^8^ molecules per cm^3^ per year and 6.5 × 10^8^ molecules per cm^3^ per year, respectively (Fig. S3†). Therefore, the trends in the model and the measurements show both a decreasing trend from 2007 to 2013 and a similar change from 2017 to 2018. Secondly, the one-year moving averages of modelled and measured monoterpene concentrations also show consistent variations, both of which show a consecutive strong peak and dip from 2010 to 2014, and a sharp increase from 2017 to 2018 (Fig. S4†). The lower trends of measurement data may result from several very low measurement concentrations around the beginning of 2012, 2017 and 2018 which were caused by low temperature during these days (not shown here).

#### 3.3.2. Long-term time series of monoterpenes

Previous studies used the empirical proxy method to investigate monoterpene seasonal and diurnal variations,^58^ which may contain high uncertainties. Based on the long-term and evaluated simulations of monoterpene concentrations by SOSAA, we analyzed the long-term yearly and seasonal trends of monoterpene emissions and concentrations at SMEAR II and presented the results in Table 1. The results show that the annual mean daily concentrations of monoterpenes increased during the last 12 years by +3.43 (+1.95 and +5.27) % per year, whereas the emission rates show only a marginal increase with negative and positive values in the 90% confidence interval and *P*_MK_ values between 0.15 and 0.23 for the three time periods. This points to a decrease in the sink terms of monoterpenes, which is mainly the reaction with OH, NO_3_ and O_3_. In the previous section, we already discussed that ozone had no significant trend in our analysis, similar to OH during daytime in summer and spring, where it has the highest impact on the monoterpene concentrations (see Section 3.4.1). However, the nitrate radical (see Section 3.4.2) decreased significantly during the last 12 years and as monoterpenes have a lifetime of hours to days, the impact of a drastically lower sink term through NO_3_ can explain the observed increase of monoterpenes.

### 3.4. Long term trends of the oxidants

#### 3.4.1. Hydroxyl radical – OH

**Fig. 5:**
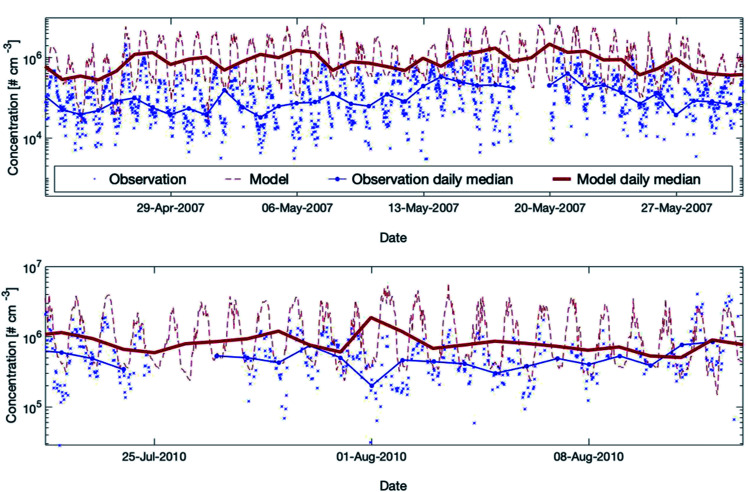
Measured *versus* modelled hydroxyl radial concentrations for two periods during the EUCAARI (upper plot) and the HUMPPA-COPEC (lower plot) campaigns. For EUCAARI, the exact measurement height is unknown, and Petäjä *et al.* (2009)^39^ mentioned that it was close to the ground level in a forest clearing. For HUMPPA-COPEC, the OH concentrations measured by CIMS (Chemical Ionization Mass Spectrometry) are used here and the measurement height was at the ground level in a different forest clearing at SMEAR II.^96^ For both campaigns, we applied here the model height level at 32.8 m assuming that the two measurement places were in the forest clearing instead of inside the forest canopy.

The OH measurements are difficult to conduct and expensive and therefore measurements of OH at SMEAR II are rare. In this study, the measurements from the EUCAARI 2007 (ref. 53) and the HUMPPA-COPEC 2010 (ref. 54) campaigns were used to evaluate the model performance. Detailed descriptions of the instruments applied for the OH measurements are provided in Kulmala *et al.* (2011)^53^ and Hens *et al.* (2014),^96^ respectively. To test the simulated OH concentration, we compared the measured data from these two campaigns against the model results (Fig. 5). Fig. S5 in the ESI† shows the scatter plots and the daily patterns of both campaigns.

During the campaign in 2007, the model overpredicted the OH concentrations substantially but the model performance showed better agreement in 2010. The main cause for the discrepancies between the two measured data sets is related to the two different seasons (May and August) during which the campaigns were conducted. The reason why the model agrees satisfactorily with the measurements in August and rather poorly in May is more complex. Additional studies comparing measured and modelled OH-reactivity at the same location^9,27,28^ showed a high missing OH-reactivity while including all measured gaseous compounds in SOSAA. These discrepancies indicate the existence of unknown compounds during springtime and early summer, which are not included in SOSAA. Preferred reactions of these species with the hydroxyl radical might explain the simulated, strong overestimation of the OH concentration at SMEAR II. Note that the uncertainty of point measurements is considerable for the boreal forest environment, as already mentioned in Section 3.3.1. The overall conclusion is that SOSAA is able to simulate the OH concentrations at SMEAR II in a sufficient way during summer but overestimates OH in spring. There are no measurements available for other seasons for comparison.

**Fig. 6:**
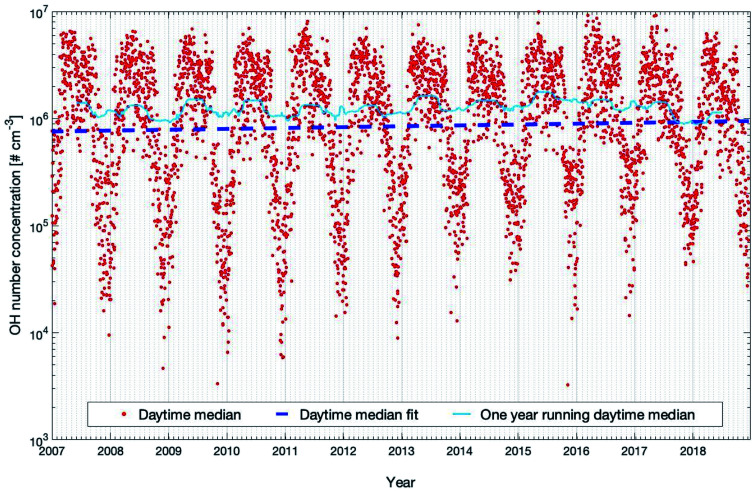
Modelled OH concentrations (20–40 m) for the years 2007 to 2018 at the SMEAR II station. Plotted are the daytime median values and the trends calculated with the linear fit and running median method which are described in detail in Section 2.3.

Fig. 6 shows the daytime time-series of modelled OH concentrations from 2007 to 2018 as OH production is related to photochemical reactions and peaks during this time of the day.^9^ The concentration shows a clear seasonal cycle with peaks in spring and late summer, which partly result from the patterns of ozone (peak in spring, see Fig. S2†) and the solar irradiance (peak in summer, see Fig. S2†), both required to produce an excited oxygen atom (O(^1^D)) which then reacts with one water molecule to form two hydroxyl radicals. Table 1 shows that OH shows significant increases in daily and nighttime trends which are +2.39 (+0.95, +3.33) % per year and 3.31 (+2.01, +4.62) % per year, respectively, with both *P*_MK_ values below 0.04. However, during daytime, the OH concentration only shows a marginal increase of +0.91 (−0.81, +2.10) % per year with a high statistical uncertainty and the *P*_MK_ value is 0.24. The trend of OH shown here is mainly related to its sink terms instead of the source terms because the main source terms of OH, *e.g.*, O_3_ and global short-wave radiation, do not show any significant increasing trends (Table 1). Among all the sink terms of OH, CO plays a major role and accounts for about 30–40% of the annual removal of OH at SMEAR II.^28,97^Table 1 shows a decreasing trend of CO which is consistent with previous studies.^98,99^ Moreover, the time series of the mixing ratio of CO in winter also shows an opposite trend to the OH concentration (Fig. S6†). However, all the decreasing daily, daytime and nighttime trends of CO are not significant (Table 1), which indicates that in order to explain the OH trend, other sink terms and their seasonal trends should be taken into account.

**Fig. 7:**
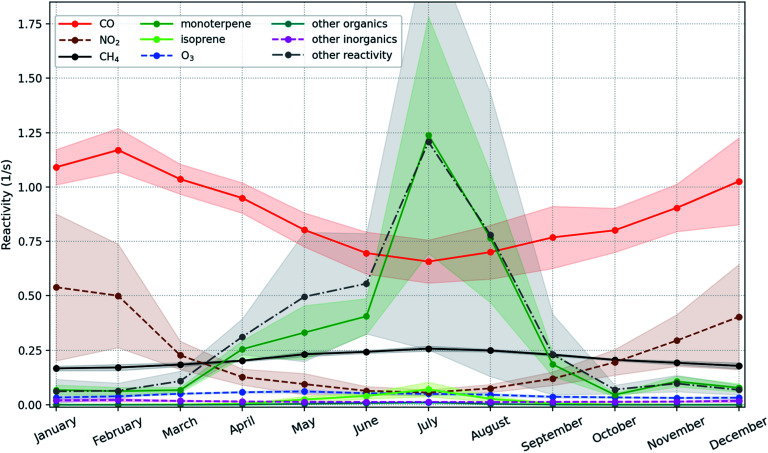
Mean monthly OH reactivity during 2007–2018 contributed by different compounds/groups with the ±1 standard deviation shown as shadows. Here “other inorganics” means all the other inorganic compounds that react with OH except the ones already plotted here. The “other organics” means all the emitted organic compounds which react with OH except isoprene and monoterpenes. The “other reactivity” means all the other organic compounds which react with OH except isoprene, monoterpenes and other organics.

Since the daytime length is shortest in winter and autumn while longest in summer and spring, the nighttime increasing trend is dominated by the trends of winter and autumn that are +10.25 (+3.95, +17.32) % per year and +4.54 (+2.39, +6.29) % per year, respectively. Fig. 7 shows that the strong increasing trends of OH in these two seasons are caused by the combined effects of decreasing CO and NO_2_. The deceasing trend of NO_2_ is much higher than that of CO with ∼9.7 times in winter and ∼5.9 times in autumn, but the OH reactivity due to NO_2_ is only about half of that due to CO (Fig. 7). The seasonal inter-annual trends of OH reactivities also show an apparent drop of R_OH,CO_ (OH reactivity due to CO) and R_OH,NO_2__ (OH reactivity due to NO_2_) during winter with R_OH,NO_2__ being a factor of 2–3 lower compared to R_OH,CO_ (Fig. S7†). Therefore, as a dominant sink of OH in winter, CO is the main factor to explain the long-term modelled OH trend, and NO_2_ also contributes a comparable portion. Moreover, monoterpenes can also produce OH *via* ozonolysis reactions, which are a main source of OH under dark conditions.^100–102^ So the increasing trend of monoterpenes could enhance the increasing nighttime trend of OH.

In spring and summer, the monoterpenes and the compounds produced from the second or higher order reactions start to be competitive with or dominant over CO among the OH reactivity contributors (Fig. 7 and S7†). The forest stands near SMEAR II are dominated by Scots pine, which produces relatively low isoprene (*e.g.*, Rinne *et al.* (2009)^26^ and references therein). For example, during summer months between 2010 and 2013 at SMEAR II, the measured flux of isoprene + MBO (2-methyl-3-buten-2-ol) was usually around one order of magnitude smaller than that of monoterpenes.^103^ This indicates that the compounds produced from the second or higher order reactions are mainly the second or higher order oxidized products of monoterpenes. Therefore, the increasing trend of monoterpenes can offset the effect of decreasing CO, leading to the insignificant daytime trends in spring and summer. And finally, a strong increasing nighttime trend and an insignificant daytime trend together lead to a significant but weaker daily trend of OH.

In the future, assuming decreasing emissions of CO and NO_2_ (change in energy production and lower nitrogen compounds of traffic emissions) and increasing monoterpene emissions in the boreal region due to climate warming,^104^ we would expect an increase of OH during winter and autumn months in central south Finland. During spring and summer, other sink terms are more relevant and OH should be buffered and remain quite constant.

#### 3.4.2. Nitrate radical – NO_3_

**Fig. 8:**
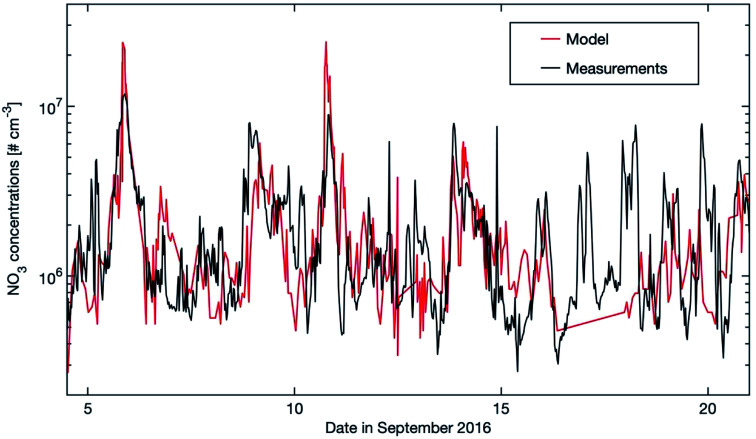
Modelled NO_3_ concentrations (20–40 m) and stationary state NO_3_ mixing ratios calculated from the production term (*k*[NO_2_][O_3_]) and using *k*_OTG_ + *k*[NO] + *J*_NO3_ as the loss term with *k*_OTG_ representing the measured reactive loss of NO_3_ to organic trace gases (Fig. 10 in Liebmann *et al.*, 2018).^56^ Measurements were obtained from the common inlet at a height of 8.5 m apart from the NO_3_ photolysis rate (taken from a height of 35 m on an adjacent tower), wind direction (WD) and wind speed (WS) (both at 16.5 m on the 128 m tower).

The nitrate radical has a very short lifetime which makes the direct measurement of NO_3_ challenging, especially in a low NO_*x*_ environment. At SMEAR II, Liebmann *et al.* (2018)^56^ tried to measure NO_3_ directly, but its mixing ratios were always below the detection limit (1.3 pptv) during the whole campaign (05.09.2016–21.09.2016). At the same time, Liebmann *et al.* (2018)^56^ measured the NO_3_ reactivity based on which they estimated the NO_3_ mixing ratios. In Fig. 8, the modelled NO_3_ mixing ratio is plotted for the same time period as in Liebmann *et al.* (2018; see Fig. 10).^56^ Our results show very low NO_3_ mixing ratios which are, similar to the campaign findings, always below 1.3 pptv. Compared to the calculated stationary state NO_3_ mixing ratios, our results are in very good agreement with the “measured” values. Moreover, both results show the peaks during the nights on the 5^th^/6^th^, 8^th^/9^th^, 10^th^/11^th^, 13^th^/14^th^ and 20^th^/21^st^ of September. By constraining SOSAA with accurately measured NO_2_ concentrations, we assume that the predicted NO_3_ concentrations are reasonable. The rapid photolysis of NO_3_ and the reaction with NO typically reduces its lifetime to a few minutes during daytime. The main contribution of the nitrate radical to the oxidation capacity of the atmosphere is during nighttime. Based on this, we will focus our analysis on the nighttime period.

**Fig. 9:**
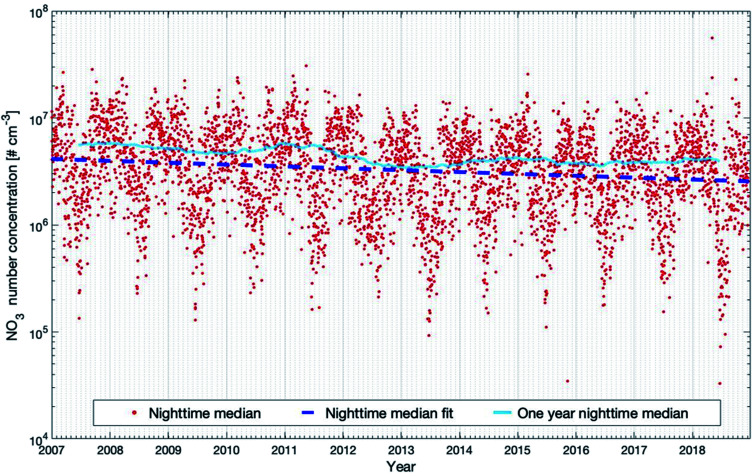
Modelled NO_3_ concentrations (20–40 m) for the years 2007 to 2018 at the SMEAR II station. Presented are the nighttime median values and the trends calculated with the linear fit and running median method which are described in detail in Section 2.3.

Fig. 9 shows the nighttime median NO_3_ concentration and the trends for the selected 12 years. The seasonal cycle shows a double-peak in late autumn and early spring, respectively. The reason is that the NO_2_ concentration is highest during these periods (see Fig. S2 in the ESI†), which may result from the high NO emissions from the microbial processes in the forest soil^105,106^ when the organic litter mass was high. As for the trend, the daytime and nighttime inter-annual trends are quite alike. The decreasing trend at night-time (−4.22 (−5.90 and −2.86) % per year) is slightly higher than the decreasing trend at daytime (−2.43 (−3.90 and −0.90) % per year). The 12 year trend of nighttime NO_3_ can be explained by the decrease of nighttime NO_2_, since the main source of NO_3_ comes from the oxidation of NO_2_ by O_3_. In Table 1, we can see that nighttime O_3_ shows no trend for the 12 years. However, nighttime NO_2_ shows a significant negative trend of −4.18 (−6.27 and −2.32) % per year, being consistent with the decreasing trend of nighttime NO_3_. The one-year running median of the nighttime NO_3_ concentration also shows an oscillation of 3–3.5 years during the years 2007–2018, but since this period is relatively short, it is hard to conclude on the reasons (Fig. 9).

### 3.5. Sulfuric acid model comparison and long-term trends

**Fig. 10:**
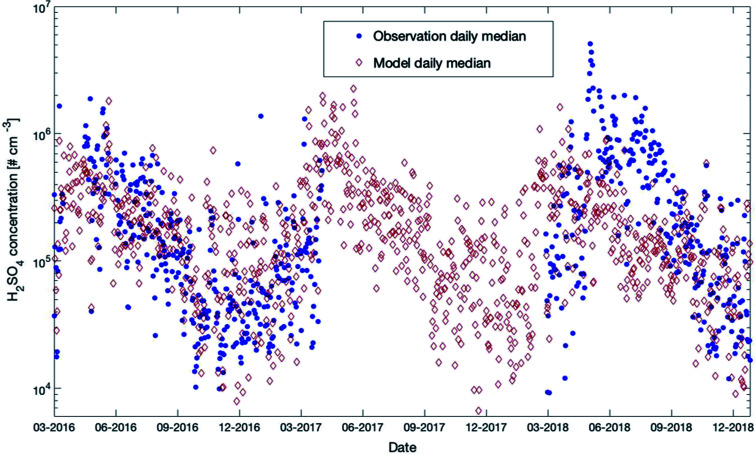
Measured (35 m) and modelled (32.8 m) daily median sulfuric acid concentrations at SMEAR II for the years 2016–2018.

Sulfuric acid was measured at SMEAR II during the last few years for several periods. In Fig. 10, we provide a comparison with the outcome of our model simulations for the years 2016 to 2018 (scatter plot and daily distributions for these data sets are provided in Fig. S8 in the ESI†). A detailed description of the instrument used in this study is provided in Jokinen *et al.* (2012).^107^

During 2016–2017, the model and measurements agreed at most times in autumn and winter. In summer 2018, the model tended to underestimate the measured concentrations but showed a very good agreement for the same year during autumn. During winter nighttime, the observations were partly below the model results and reached values down to a couple of hundreds of molecules per cm^3^. However, the LOD of the instrument was 4 × 10^4^ molecules cm^−3^ (ref. 107) and most of the measurements during that period were below the LOD. This could explain why the daily median values in winter were lower compared to the simulated values.

**Fig. 11:**
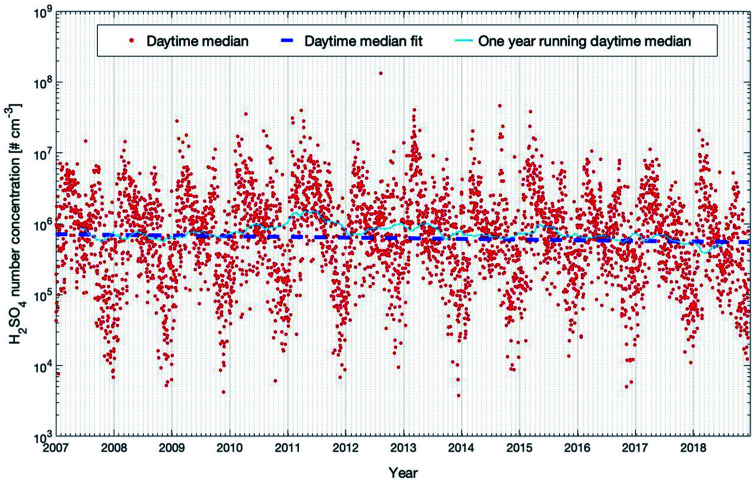
Modelled sulfuric acid concentrations between 20 m and 40 m for the years 2007 to 2018 at the SMEAR II station. Shown are the daytime median values and the trends calculated using the linear fit and running median which are described in detail in Section 2.3.

Both the model and the observations present an interesting pattern for the three years: a peak in early spring and then a continuous decline of concentrations for the rest of the year. There is a smaller second peak in summers visible in the model data set, but this peak is weaker compared to the spring peak, as can also be seen in Fig. 11, which provides the modelled 12-year daytime median concentrations of sulfuric acid. The reason for this pattern, which is visible in the model outcomes for all years, is a combination of mainly three effects: SO_2_, one of the two main precursors of H_2_SO_4_, peaks in late winter and early spring and OH reaches its yearly maximum in spring. Additionally, the condensation sink, representing the rate of how fast sulfuric acid molecules will condense on the existing particles, has a clear maximum in summer (Fig. S2 in the ESI†). These three parameters are mainly responsible for the sulfuric acid pattern. Note that a similar pattern has been observed for the occurrence of NPF events at SMEAR II for several years.^90^

Nieminen *et al.* (2014)^90^ predicted the trend of sulfuric acid based on a proxy calculation (see the next section) with −1.3% on NPF days and −0.3% on non-NPF days per year for the years 1997–2012. In our study, we applied SOSAA simulations for the years 2007–2018 for the same location. Our model results predict a stronger decrease of daytime H_2_SO_4_ of −2.78 (−6.05 and −0.63) % per year (see Table 1). However, the confidence interval of this trend is quite broad and the *P*_MK_ value is 0.18, which suggests that caution should be taken to interpret this trend too far to the future. The trend in the studied time span is greatly influenced by the large yearly variation. With decreasing emission of SO_2_ in the future (related to improved filter systems), we can expect a decreasing trend of H_2_SO_4_ in central southern Finland which could have a significant impact on the amount of newly formed particles and consequently the number concentrations of cloud condensation nuclei.

### 3.6. Proxy comparison for OH, H_2_SO_4_ and NO_3_

During the last few years, several proxies have been developed for compounds such as the hydroxyl or nitrate radicals due to the absence or sparse long-term observations for these parameters. In this section, we will compare some of these proxies with the outcome of our model simulations for SMEAR II. This, however, should not be seen as a validation of the proxies but rather to investigate how well the simulations agree with them. The proxies compared were developed based on datasets from SMEAR II.

**Fig. 12:**
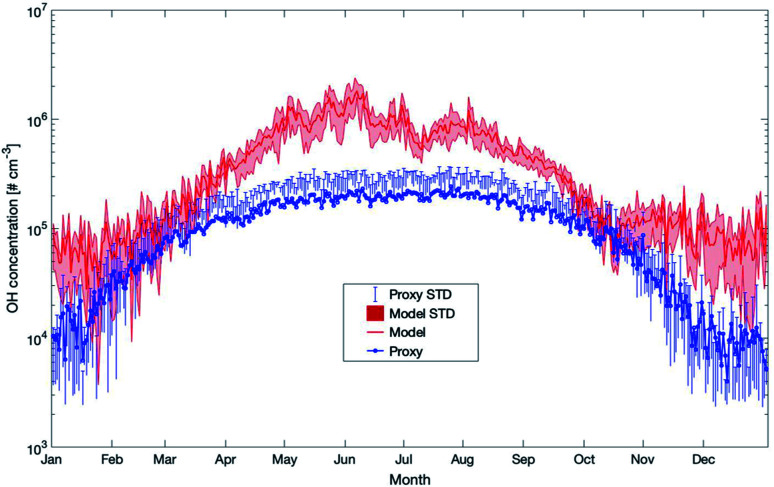
Yearly mean daily time series of OH concentrations estimated by SOSAA (model) at 32.8 m and a proxy parameterization (proxy) for the years 2007–2018 at SMEAR II. Details of the applied proxy are provided in the text (STD = standard deviation).

The first proxy we compare is for the OH radical. It is based on Petäjä *et al.* (2009)^39^ and Nieminen *et al.* (2014).^90^ The results of the proxy, together with the outcome of the model simulations, are presented in Fig. 12. In this proxy, the hydroxyl radical is calculated as

1 [OH] = ((8.4 × 10^−7^/8.6 × 10^−10^) × UVB^0.32^)^1.92^

Here UVB is the ultraviolet irradiance measured at SMEAR II. The modelled and the proxy OH concentrations show similar values in February and at the beginning of October but differ quite strongly during the rest of the year. The modelled values increase much stronger in early spring and reach a maximum in May. At this time, the discrepancy between the proxy and the model reaches up to one order of magnitude and decreases afterwards. As pointed out in Section 3.4.1, there exist missing compound(s) reacting with OH which have not been identified until now. Taking this into account and assuming that the missing compounds originate from the local ecosystem with maximum emissions during the most biologically active period, the modelled OH concentrations are potentially too high during spring and early summer. However, we assume that the missing OH-reactivity would not explain the one order difference during spring and summer between the model and the proxy. Rather, we would expect that the contribution of OH production throughout the ozonolysis of terpenes is a missing factor in the proxies as the proxy is only based on UVB measurements. As this proxy was constructed when the HUMPPA-COPEC 2010 (ref. 54) campaign measured OH concentrations from 2010 and the monoterpene concentrations have increased significantly since then (see Section 3.3.2), a certain fraction of the OH difference between the model and the proxy could be related to this mechanism and would require constructing a new proxy with up-to-date OH measurements.

Later in the year, from October to February, the proxy showed very low OH values related to the main and only source by UVB. In reality, the ozonolysis of terpenes will contribute to the OH-concentration at this time and the model data seems to be more realistic during periods with low photolysis rates. However, as long as these unknown compounds are not identified and no long-term measurements of OH at SMEAR II exist, any final conclusion about whether the proxy or the model is more correct can only be speculation. However, based on our previous OH-reactivity studies, we are confident that certain crucial reactions are still missing. For this reason, the simplified relation between UVB and OH could provide a more realistic picture on the hydroxyl radical concentrations during spring and early summer.

The next proxy we compare with our model simulations is for sulfuric acid. It is based on a new parameterization method from Dada *et al.* (2020).^108^ This proxy is calculated as follows:

2 [H_2_SO_4_] = −CS/2 × *k*_3_ + ((CS/2 × *k*_3_)^2^ + [SO_2_]/*k*_3_ × (*k*_2_ × GloRad + *k*_2_ × [O_3_] × [alkene]))^0.5^.

Here GloRad stands for global irradiance, CS for the condensational sink, and [SO_2_] and [alkene] for the gas phase concentrations of sulfur dioxide and the sum of monoterpenes, respectively. The coefficient *k*_1_ (0.85 × 10^−8^ m^2^ W^−1^ s^−1^) represents the coefficient of H_2_SO_4_ production due to the SO_2_–OH reaction; *k*_2_ (6.1 × 10^−29^ cm^6^ s^−1^) is the coefficient of H_2_SO_4_ production *via* the stabilized Criegee intermediates produced by the ozonolysis of alkenes and *k*_3_ (4.26 × 10^−9^ cm^3^ s^−1^) is the clustering coefficient for the square of the sulfuric acid concentrations, which takes into account the loss of H_2_SO_4_ due to cluster formation, not included in the condensation sink term.

**Fig. 13:**
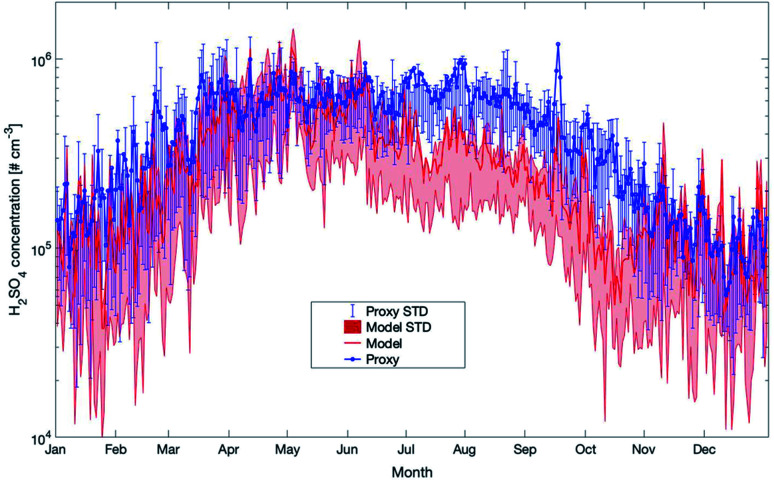
Yearly mean daily time series of H_2_SO_4_ concentrations estimated by SOSAA (model) and a proxy parameterization (proxy) at 32.8 m for the years 2007–2018 at SMEAR II. Details of the applied proxy are provided in the text (STD = standard deviation).

As already pointed out in the previous section, the modelled sulfuric acid concentrations show a clear peak in early spring and then a nearly continuous decrease for the rest of the year (see Fig. 13). The proxy follows this pattern throughout the year but exceeds the modelled data by a factor of 2 from the end of June to the end of October and during some shorter periods in winter. As the measured values for these periods seem to agree well with the model results, we conclude that the proxy in summer and autumn overestimates the H_2_SO_4_ concentrations and we assume that for this parameter, the model provides a more realistic picture of the sulfuric acid concentrations.

The last proxy we want to compare against the model results is for the nitrate radical. It is based on Peräkylä *et al.* (2014)^57^ and Kontkanen *et al.* (2016).^58^ The concentration of NO_3_ is calculated based on the following equation:

3 [NO_3_] = *k*_O_3_+NO_2__ × [O_3_] × [NO_2_] × *τ*_NO_3__

Here *k*_O_3_+NO_2__ is the temperature-dependent reaction rate coefficient between NO_2_ and O_3_, which was calculated from a temperature-dependent relation^109^ (see Table A1† in Kontkanen *et al.*, 2016 (ref. 58)). *τ*_NO_3__ is the lifetime of NO_3_ and a detailed description of the prediction of *τ*_NO_3__ is available in the manuscript by Kontkanen *et al.* (2016).^58^

**Fig. 14:**
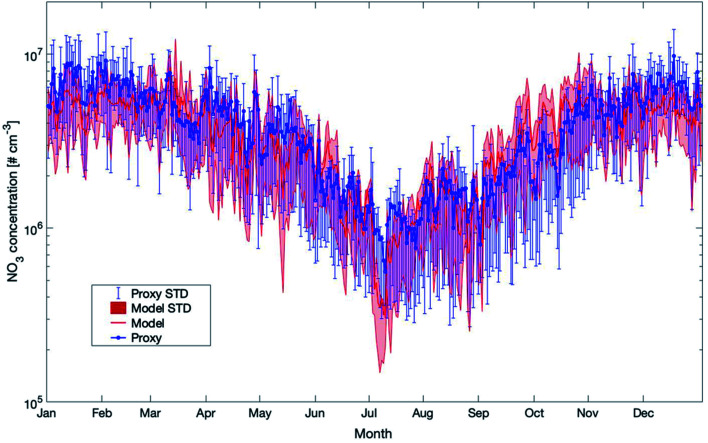
Yearly mean daily time series of NO_3_ concentrations estimated by SOSAA (Model) and a proxy parameterization (Proxy) at 32.8 m based on the reference for the years 2007–2018 at SMEAR II (STD = standard deviation).

Previous studies assumed a steady state between the production of NO_3_ from the reaction between O_3_ and NO_2_ and the removal of NO_3_.^57,58^ This gap could be filled by our study. A comparison between the proxy and the model data shows that the long-term trend from both methods is in very good agreement (Fig. 14). As these two methods applied here agree very well, it is likely that the predicted values for NO_3_ are reliable and could be applied in further studies.

## 4. Summary and perspectives

In this study, we investigated the trends of selected atmospherically crucial gaseous compounds at the SMEAR II station in southern Finland for the period 2007–2018. The main focus was on the hydroxyl and nitrate radicals as well as on sulfuric acid, as no long-term measurements of these compounds exist. To validate the SOSAA model, we first compared the OH, H_2_SO_4_ and NO_3_ simulations with the existing measurements from several campaigns. For H_2_SO_4_, the model underestimated the measured values in summer 2018 but reproduced the measurements for the same period in 2016. For all other seasons, the model and the measurements agree satisfactorily. The OH radical was only measured during two short campaigns in May 2007 (ref. 53) and August 2010.^54^ The comparison between the observed and modelled OH yielded different results in the campaigns of May 2007 and August 2010. In 2007 the model predicted about twice as high values as measured whereas in 2010 the model agreed quite well with the measurements, reflecting the existence of an unknown sink(s) for OH in spring and early summer. In the case of the nitrate radical, no direct measurements at SMEAR II exist. However, Liebmann *et al.* (2018)^56^ measured the NO_3_ reactivity based on which they estimated the NO_3_ mixing ratios. The comparison of the indirect NO_3_ measurements with our model results showed a good agreement for the selected short period in September 2016.

The long-term trends (12 years) of the two important oxidants OH and NO_3_ were investigated. Our results indicate that the daily OH concentration increases during this period with a rate of +2.38 (+0.95, +3.33) % per year, which relates to a significant increase of OH during winter nighttime (+10.66 (+5.04, 17.37) % per year). The main reason is likely a drop in carbon monoxide concentrations (∼0.5% per year) during winter representing the main sink term for OH during this period. This result was surprising as the monoterpenes, the main biogenic VOC at SMEAR II, increased by about +3.40 (+1.23, +6.02) % per year and reacted strongly with OH. Therefore, the predicted OH trend shows that the climatic temperature increases (∼0.8 K in 12 years at SMEAR II) and the following rise in BVOC emissions is buffered by a decline of carbon monoxide in winter months. In case the current negative trend in CO continues – mostly related to improved combustion techniques – OH will slightly rise if monoterpene concentrations do not increase more significantly. This would cause a positive impact on the atmospheric oxidation capacity. *Vice versa* is the situation for NO_3_, showing a nighttime decrease by −4.22 (−5.90, −2.86) % per year which is caused by the drop in nitrogen dioxide. As all anthropogenic NO_*x*_ emissions in Europe have decreased significantly during the last few decades (EAA report No 12/2018) and are predicted to decrease further, we expect that the nitrate radical will continue to drop in the future. As pointed out by Mogensen *et al.* (2015),^9^ NO_3_ is the strongest oxidant during nighttime and can have an aerosol yield when reacting with monoterpenes up to 65%.^110^ A continuous drop in NO_3_ in the boreal forest could implicate a negative impact on the growth of SOA during nighttime and decrease in the CCN concentrations.

Sulfuric acid was investigated as it is one of the most important precursors of new particle formation (NPF). The outcome of our study indicates that the sulfuric acid concentration decreases by −2.78 (−6.05 and −0.63) % per year during daytime, which likely is related to the reduction in the emissions of sulfur dioxide in Europe during the last few decades (EAA report No 12/2018). In case the negative trend of sulfuric acid (steered by SO_2_) will continue in the next few decades, it could significantly affect the amount of NPF events in the boreal region. However, whether or not this will have a positive or negative impact on our future climate is currently unclear. In the past, it was typically assumed that NPF events will provide more CCN, followed by more cloud droplets, leading to an increased albedo through “brighter” clouds.^111^ In this way, NPF would cool the planet and counteract the effect of greenhouse gases. However, recently the research results of Roldin *et al.* (2019)^15^ counteract this assumption. Their results showed that under some meteorological conditions a high number of newly formed particles increase in size at the expense of the larger aerosol particles over the boreal forest – and it is only the larger aerosol particles that have a cooling effect on the planet. Facing the controversial discussion on this topic in the scientific community, it is difficult to state whether the decrease of sulfuric acid should be seen as positive or negative. However, it is certain that H_2_SO_4_ has decreased in the last few decades and, most likely, will continue to drop in the future.

Proxies are commonly applied in case a limited number of parameters are measured and no detailed model simulations are available. We compared the concentrations of OH, NO_3_ and H_2_SO_4_ calculated from proxies^39,57,58,90,108^ with our model outcomes. Our comparisons showed that the proxies for OH and H_2_SO_4_, at certain times of the year, agree very well with the model results but also differ significantly during other periods. For the nitrate radical, the model and proxy results are in good agreement.

## Special issue statement

This article is part of the special issue “Pan-Eurasian Experiment (PEEX)”. It is not associated with a conference.

## Data availability

All data shown in the figures, tables and additional raw data are available upon request from the corresponding author (PZ).

## Author contributions

M. Boy and PZ conceived the idea and coordinated the work. DC performed the model simulations, wrote main parts of the manuscript and plotted Fig. 1 to 6, 8 to 14, S1, S2, S5 and S8.† CX, PC and TN implemented the statistical analysis of the data and plotted Fig. 7, S3, S4, S6 and S7.† PZ, M. Boy, P. Roldin and XQ were the main model developers and assisted in setting up the model for the simulations. P. Rantala, JA, NS, P. Kolari and P. Keronen provided the input data for the model runs and for the model evaluation. LP, MK, MPR, DT, BF and M. Baykara contributed significantly to the data interpretation of the model. All co-authors commented on the paper and supported the finalization of the manuscript. PZ oversaw the responses to the reviewer comments.

## Conflicts of interest

The authors declare that they have no conflict of interest.

## Supplementary Material

EA-001-D1EA00020A-s001

## References

[cit1] Gligorovski S., Strekowski R., Barbati S., Vione D. (2015). Environmental Implications of Hydroxyl Radicals (˙OH). Chem. Rev..

[cit2] Bey I., Aumont B., Toupance G. (2001). A modeling study of the nighttime radical chemistry in the lower continental troposphere: 1. Development of a detailed chemical mechanism including nighttime chemistry. J. Geophys. Res., D: Atmos..

[cit3] Allan B. J., Plane J. M. C., Coe H., Shillito J. (2002). Observations of NO_3_ concentration profiles in the troposphere. J. Geophys. Res..

[cit4] Brown S. S., Stark H., Ryerson T. B., Williams E. J., Nicks D. K., Trainer M., Fehsenfeld F. C., Ravishankara A. R. (2003). Nitrogen oxides in the nocturnal boundary layer: simultaneous *in situ* measurements of NO_3_, N_2_O_5_, NO_2_, NO, and O_3_. J. Geophys. Res., D: Atmos..

[cit5] Crowley J. N., Schuster G., Pouvesle N., Parchatka U., Fischer H., Bonn B., Bingemer H., Lelieveld J. (2010). Nocturnal nitrogen oxides at a rural mountain-site in south-western Germany. Atmos. Chem. Phys..

[cit6] Elshorbany Y. F., Kurtenbach R., Wiesen P., Lissi E., Rubio M., Villena G., Gramsch E., Rickard A. R., Pilling M. J., Kleffmann J. (2009). Oxidation capacity of the city air of Santiago, Chile. Atmos. Chem. Phys..

[cit7] Volkamer R., Sheehy P., Molina L. T., Molina M. J. (2010). Oxidative capacity of the Mexico City atmosphere – Part 1: a radical source perspective. Atmos. Chem. Phys..

[cit8] Mao J., Ren X., Chen S., Brune W. H., Chen Z., Martinez M., Harder H., Lefer B., Rappenglück B., Flynn J., Leuchner M. (2010). Atmospheric oxidation capacity in the summer of Houston 2006: comparison with summer measurements in other metropolitan studies. Atmos. Environ..

[cit9] Mogensen D., Gierens R., Crowley J. N., Keronen P., Smolander S., Sogachev A., Nölscher A. C., Zhou L., Kulmala M., Tang M. J., Williams J., Boy M. (2015). Simulations of atmospheric OH, O_3_ and NO_3_ reactivities within and above the boreal forest. Atmos. Chem. Phys..

[cit10] Feiner P. A., Brune W. H., Miller D. O., Zhang L., Cohen R. C., Romer P. S., Goldstein A. H., Keutsch F. N., Skog K. M., Wennberg P. O., Nguyen T. B., Teng A. P., DeGouw J., Koss A., Wild R. J., Brown S. S., Guenther A., Edgerton E., Baumann K., Fry J. L. (2016). Testing Atmospheric Oxidation in an Alabama Forest. J. Atmos. Sci..

[cit11] Stone D., Evans M. J., Walker H., Ingham T., Vaughan S., Ouyang B., Kennedy O. J., McLeod M. W., Jones R. L., Hopkins J., Punjabi S., Lidster R., Hamilton J. F., Lee J. D., Lewis A. C., Carpenter L. J., Forster G., Oram D. E., Reeves C. E., Bauguitte S., Morgan W., Coe H., Aruffo E., Dari-Salisburgo C., Giammaria F., Di Carlo P., Heard D. E. (2014). Radical chemistry at night: comparisons between observed and modelled HO_*x*_, NO_3_ and N_2_O_5_ during the RONOCO project. Atmos. Chem. Phys..

[cit12] Bonn B., von Kuhlmann R., Lawrence M. G. (2004). High contribution of biogenic hydroperoxides to secondary organic aerosol formation. Geophys. Res. Lett..

[cit13] Claeys M., Graham B., Vas G., Wang W., Vermeylen R., Pashynska V., Cafmeyer J., Guyon P., Andreae M. O., Artaxo P., Maenhaut W. (2004). Formation of secondary organic aerosols through photooxidation of isoprene. Science.

[cit14] Hallquist M., Wenger J. C., Baltensperger U., Rudich Y., Simpson D., Claeys M., Dommen J., Donahue N. M., George C., Goldstein A. H., Hamilton J. F., Herrmann H., Hoffmann T., Iinuma Y., Jang M., Jenkin M. E., Jimenez J. L., Kiendler-Scharr A., Maenhaut W., McFiggans G., Mentel T. F., Monod A., Prévôt A. S. H., Seinfeld J. H., Surratt J. D., Szmigielski R., Wildt J. (2009). The formation, properties and impact of secondary organic aerosol: current and emerging issues. Atmos. Chem. Phys..

[cit15] Roldin P., Ehn M., Kurtén T., Olenius T., Rissanen M. P., Sarnela N., Elm J., Rantala P., Hao L., Hyttinen N., Heikkinen L., Worsnop D. R., Pichelstorfer L., Xavier C., Clusius P., Öström E., Petäjä T., Kulmala M., Vehkamäki H., Virtanen A., Riipinen I., Boy M. (2019). The role of highly oxygenated organic molecules in the Boreal aerosol-cloud-climate system. Nat. Commun..

[cit16] Riccobono F., Schobesberger S., Scott C. E., Dommen J., Ortega I. K., Rondo L., Almeida J., Amorim A., Bianchi F., Breitenlechner M., David A., Downard A., Dunne E. M., Duplissy J., Ehrhart S., Flagan R. C., Franchin A., Hansel A., Junninen H., Kajos M., Keskinen H., Kupc A., Kurten A., Kvashin A. N., Laaksonen A., Lehtipalo K., Makhmutov V., Mathot S., Nieminen T., Onnela A., Petäjä T., Praplan A. P., Santos F. D., Schallhart S., Seinfeld J. H., Sipila M., Spracklen D. V., Stozhkov Y., Stratmann F., Tome A., Tsagkogeorgas G., Vaattovaara P., Viisanen Y., Vrtala A., Wagner P. E., Weingartner E., Wex H., Wimmer D., Carslaw K. S., Curtius J., Donahue N. M., Kirkby J., Kulmala M., Worsnop D. R., Baltensperger U. (2014). Oxidation Products
of Biogenic Emissions Contribute to Nucleation of Atmospheric Particles. Science.

[cit17] Kirkby J., Duplissy J., Sengupta K., Frege C., Gordon H., Williamson C., Heinritzi M., Simon M., Yan C., Almeida J., Tröstl J., Nieminen T., Ortega I. K., Wagner R., Adamov A., Amorim A., Bernhammer A.-K., Bianchi F., Breitenlechner M., Brilke S., Chen X., Craven J., Dias A., Ehrhart S., Flagan R. C., Franchin A., Fuchs C., Guida R., Hakala J., Hoyle C. R., Jokinen T., Junninen H., Kangasluoma J., Kim J., Krapf M., Kürten A., Laaksonen A., Lehtipalo K., Makhmutov V., Mathot S., Molteni U., Onnela A., Peräkylä O., Piel F., Petäjä T., Praplan A. P., Pringle K., Rap A., Richards N. A. D., Riipinen I., Rissanen M. P., Rondo L., Sarnela N., Schobesberger S., Scott C. E., Seinfeld J. H., Sipilä M., Steiner G., Stozhkov Y., Stratmann F., Tomé A., Virtanen A., Vogel A. L., Wagner A. C., Wagner P. E., Weingartner E., Wimmer D., Winkler P. M., Ye P., Zhang X., Hansel A., Dommen J., Donahue N. M., Worsnop D. R., Baltensperger U., Kulmala M., Carslaw K. S., Curtius J. (2016). Ion-induced nucleation of pure biogenic particles. Nature.

[cit18] Gordon H., Kirkby J., Baltensperger U., Bianchi F., Breitenlechner M., Curtius J., Dias A., Dommen J., Donahue N. M., Dunne E. M., Duplissy J., Ehrhart S., Flagan R. C., Frege C., Fuchs C., Hansel A., Hoyle C. R., Kulmala M., Kürten A., Lehtipalo K., Makhmutov V., Molteni U., Rissanen M. P., Stozkhov Y., Tröstl J., Tsagkogeorgas G., Wagner R., Williamson C., Wimmer D., Winkler P. M., Yan C., Carslaw K. S. (2017). Causes and importance of new particle formation in the present-day and preindustrial atmospheres. J. Geophys. Res.: Atmos..

[cit19] Dunne E. M., Gordon H., Kürten A., Almeida J., Duplissy J., Williamson C., Ortega I. K., Pringle K. J., Adamov A., Baltensperger U., Barmet P., Benduhn F., Bianchi F., Breitenlechner M., Clarke A., Curtius J., Dommen J., Donahue N. M., Ehrhart S., Flagan R. C., Franchin A., Guida R., Hakala J., Hansel A., Heinritzi M., Jokinen T., Kangasluoma J., Kirkby J., Kulmala M., Kupc A., Lawler M. J., Lehtipalo K., Makhmutov V., Mann G., Mathot S., Merikanto J., Miettinen P., Nenes A., Onnela A., Rap A., Reddington C. L. S., Riccobono F., Richards N. A. D., Rissanen M. P., Rondo L., Sarnela N., Schobesberger S., Sengupta K., Simon M., Sipilä M., Smith J. N., Stozkhov Y., Tomé A., Tröstl J., Wagner P. E., Wimmer D., Winkler P. M., Worsnop D. R., Carslaw K. S. (2016). Global atmospheric particle formation from CERN CLOUD measurements. Science.

[cit20] Guenther A. B., Hewitt C., Erickson D., Fall R., Geron C., Graedel T., Harley P., Klinger L., Lerdau M., McKay W., Pierce T., Scholes R., Steinbrecher R., Tallamraju R., Taylor J., Zimmerman P. (1995). A global model of natural volatile organic compound emissions. J. Geophys. Res..

[cit21] Guenther A. B., Karl T., Harley P., Wiedinmyer C., Palmer P. I., Geron C. (2006). Estimates of global terrestrial isoprene emissions using MEGAN (Model of Emissions of Gases and Aerosols from Nature). Atmos. Chem. Phys..

[cit22] Bonan G. B. (2008). Forests and Climate Change: Forcings, Feedbacks and the Climate Benefits of Forests. Science.

[cit23] FAO , Global Forest Resources Assessment 2015, UN Food and Agriculture Organization, Rome, 2015

[cit24] Hakola H., Rinne J., Laurila T. (1998). The hydrocarbon emission rates of tea-leafed willow (Salix phylicifolia), silver birch (Betula pendula) and European aspen (Populus tremula). Atmos. Environ..

[cit25] Hakola H., Tarvainen V., Bäck J., Ranta H., Bonn B., Rinne J., Kulmala M. (2006). Seasonal variation of mono- and sesquiterpene emission rates of Scots pine. Biogeosciences.

[cit26] Rinne J., Bäck J., Hakola H. (2009). Biogenic volatile organic compound emissions from the Eurasian taiga: current knowledge and future directions. Boreal Environ. Res..

[cit27] Mogensen D., Smolander S., Sogachev A., Zhou L., Sinha V., Guenther A., Williams J., Nieminen T., Kajos M. K., Rinne J., Kulmala M., Boy M. (2011). Modelling atmospheric OH-reactivity in a boreal forest ecosystem. Atmos. Chem. Phys..

[cit28] Praplan A. P., Tykkä T., Chen D., Boy M., Taipale D., Vakkari V., Zhou P., Petäjä T., Hellén H. (2019). Long-term total OH reactivity measurements in a boreal forest. Atmos. Chem. Phys..

[cit29] Wilson R. C., Fleming Z. L., Monks P. S., Clain G., Henne S., Konovalov I. B., Szopa S., Menut L. (2012). Have primary emission reduction measures reduced ozone across Europe? An analysis of European rural background ozone trends 1996–2005. Atmos. Chem. Phys..

[cit30] Yan Y., Pozzer A., Ojha N., Lin J., Lelieveld J. (2018). Analysis of European ozone trends in the period 1995–2014. Atmos. Chem. Phys..

[cit31] Montzka S. A., Spivakovsky C. M., Butler J. H., Elkins J. W., Lock L. T., Mondeel D. J. (2000). New observational constraints for atmospheric hydroxyl on global and hemispheric scales. Science.

[cit32] Prinn R. G., Huang J., Weiss R. F., Cunnold D. M., Fraser P. J., Simmonds P. G., McCulloch A., Harth C., Salameh P., O'Doherty S., Wang R. H. J., Porter L., Miller B. R. (2001). Evidence for substantial variations of atmospheric hydroxyl radicals in the past two decades. Science.

[cit33] Kirschke S., Bousquet P., Ciais P., Saunois M., Canadell J. G., Dlugokencky E. J., Bergamaschi P., Bergmann D., Blake D. R., Bruhwiler L., Cameron-Smith P., Castaldi S., Chevallier F., Feng L., Fraser A., Heimann M., Hodson E. L., Houweling S., Josse B., Fraser P. J., Krummel P. B., Lamarque J.-F., Langenfelds R. L., Le Quéré C., Naik V., O'Doherty S., Palmer P. I., Pison I., Plummer D., Poulter B., Prinn R. G., Rigby M., Ringeval B., Santini M., Schmidt M., Shindell D. T., Simpson I. J., Spahni R., Steele L. P., Strode S. A., Sudo K., Szopa S., van der Werf G. R., Voulgarakis A., van Weele M., Weiss R. F., Williams J. E., Zeng G. (2013). Three decades of global methane sources and sinks. Nat. Geosci..

[cit34] Montzka S. A., Krol M., Dlugokencky E., Hall B., Jöckel P., Lelieveld J. (2011). Small interannual variability of global atmospheric hydroxyl. Science.

[cit35] Rohrer F., Berresheim H. (2006). Strong correlation between levels of tropospheric hydroxyl radicals and solar ultraviolet radiation. Nature.

[cit36] Heintz F., Platt U., Flentje H., Dubois R. (1996). Long-term observation of nitrate radicals at the Tor Station, Kap Arkona (Rügen). J. Geophys. Res., D: Atmos..

[cit37] Eisele F. L., Tanner D. J. (1991). Ion-assisted tropospheric OH measurements. J. Geophys. Res..

[cit38] Holland F., Hessling M., Hofzumahaus A. (1991). In Situ Measurement of Tropospheric OH Radicals by Laser-Induced Fluorescence – A Description of the KFA Instrument. J. Atmos. Sci..

[cit39] Petäjä T., Mauldin III R. L., Kosciuch E., McGrath J., Nieminen T., Paasonen P., Boy M., Adamov A., Kotiaho T., Kulmala M. (2009). Sulfuric acid and OH concentrations in a boreal forest site. Atmos. Chem. Phys..

[cit40] Tan D., Faloona I., Simpas J., Brune W., Shepson P., Couch T. L., Sumner A., Carroll M., Thornberry T., Apel E., Riemer D., Stockwell W. (2001). HO_*x*_ budgets in a deciduous forest: results from the PROPHET summer 1998 campaign. J. Geophys. Res.: Atmos..

[cit41] Ren X., Brune W., Cantrell C., Edwards G., Shirley T., Metcalf A., Lesher R. (2005). Hydroxyl and peroxy radical chemistry in a rural area of central pennsylvania: observations and model comparisons. J. Atmos. Chem..

[cit42] Kubistin D., Harder H., Martinez M., Rudolf M., Sander R., Bozem H., Eerdekens G., Fischer H., Gurk C., Klüpfel T., Königstedt R., Parchatka U., Schiller C. L., Stickler A., Taraborrelli D., Williams J., Lelieveld J. (2010). Hydroxyl radicals in the tropical troposphere over the Suriname rainforest: comparison of measurements with the box model MECCA. Atmos. Chem. Phys..

[cit43] Dlugi R., Berger M., Zelger M., Hofzumahaus A., Siese M., Holland F., Wisthaler A., Grabmer W., Hansel A., Koppmann R., Kramm G., Möllmann-Coers M., Knaps A. (2010). Turbulent exchange and segregation of HO_*x*_ radicals and volatile organic compounds above a deciduous forest. Atmos. Chem. Phys..

[cit44] Kanaya Y., Hofzumahaus A., Dorn H.-P., Brauers T., Fuchs H., Holland F., Rohrer F., Bohn B., Tillmann R., Wegener R., Wahner A., Kajii Y., Miyamoto K., Nishida S., Watanabe K., Yoshino A., Kubistin D., Martinez M., Rudolf M., Harder H., Berresheim H., Elste T., PlassDülmer C., Stange G., Kleffmann J., Elshorbany Y., Schurath U. (2012). Comparisons of observed and modeled OH and HO_2_ concentrations during the ambient measurement period of the HO*x* Comp field campaign. Atmos. Chem. Phys..

[cit45] Regelin E., Harder H., Martinez M., Kubistin D., Tatum Ernest C., Bozem H., Klippel T., Hosaynali-Beygi Z., Fischer H., Sander R., Jöckel P., Königstedt R., Lelieveld J. (2013). HO_*x*_ measurements in the summertime upper troposphere over Europe: a comparison of observations to a box model and a 3-D model. Atmos. Chem. Phys..

[cit46] Heard D. E., Carpenter L. J., Creasey D. J., Hopkins J. R., Lee J. D., Lewis A. C., Pilling M. J., Seakins P. W., Carslaw N., Emmerson K. M. (2004). High levels of the hydroxyl radical in the winter urban troposphere. Geophys. Res. Lett..

[cit47] Emmerson K., Carslaw N., Carpenter L., Heard D., Lee J. D., Pilling M. (2005). Urban Atmospheric Chemistry During the PUMA Campaign 1: Comparison of Modelled OH and HO_2_ Concentrations with Measurements. J. Atmos. Chem..

[cit48] Shirley T. R., Brune W. H., Ren X., Mao J., Lesher R., Cardenas B., Volkamer R., Molina L. T., Molina M. J., Lamb B., Velasco E., Jobson T., Alexander M. (2006). Atmospheric oxidation in the Mexico City Metropolitan Area (MCMA) during April 2003. Atmos. Chem. Phys..

[cit49] Martinez M., Harder H., Kovacs T. A., Simpas J. B., Bassis J., Lesher R., Brune W. H., Frost G. J., Williams E. J., Stroud C. A., Jobson B. T., Roberts J. M., Hall S. R., Shetter R. E., Wert B., Fried A., Alicke B., Stutz J., Young V. L., White A. B., Zamora R. J. (2003). OH and HO_2_ concentrations, production and loss rates during the Southern Oxidant Study in Nashville, TN, summer 1999. J. Geophys. Res..

[cit50] Emmerson K. M., Carslaw N., Carslaw D. C., Lee J. D., McFiggans G., Bloss W. J., Gravestock T., Heard D. E., Hopkins J., Ingham T., Pilling M. J., Smith S. C., Jacob M., Monks P. S. (2007). Free radical modelling studies during the UK TORCH campaign in summer 2003. Atmos. Chem. Phys..

[cit51] Griffith S. M., Hansen R. F., Dusanter S., Michoud V., Gilman J. B., Kuster W. C., Veres P. R., Graus M., de Gouw J. A., Roberts J., Young C., Washenfelder R., Brown S. S., Thalman R., Waxman E., Volkamer R., Tsai C., Stutz J., Flynn J. H., Grossberg N., Lefer B., Alvarez S. L., Rappenglueck B., Mielke L. H., Osthoff H. D., Stevens P. S. (2016). Measurements of hydroxyl and hydroperoxy radicals during CalNex-LA: model comparisons and radical budgets. J. Geophys. Res.: Atmos..

[cit52] Kulmala M., Hämeri K., Aalto P. P., Mäkelä J. M., Pirjola L., Nilsson E. D., Buzorius G., Rannik Ü., Maso M. D., Seidl W., Hoffman T., Janson R., Hansson H.-C., Viisanen Y., Laaksonen A., O'dowd C. D. (2001). Overview of the international project on biogenic aerosol formation in the boreal forest (BIOFOR). Tellus B.

[cit53] Kulmala M., Asmi A., Lappalainen H. K., Baltensperger U., Brenguier J.-L., Facchini M. C., Hansson H.-C., Hov Ø., O'Dowd C. D., Pöschl U., Wiedensohler A., Boers R., Boucher O., de Leeuw G., Denier van der Gon H. A. C., Feichter J., Krejci R., Laj P., Lihavainen H., Lohmann U., McFiggans G., Mentel T., Pilinis C., Riipinen I., Schulz M., Stohl A., Swietlicki E., Vignati E., Alves C., Amann M., Ammann M., Arabas S., Artaxo P., Baars H., Beddows D. C. S., Bergström R., Beukes J. P., Bilde M., Burkhart J. F., Canonaco F., Clegg S. L., Coe H., Crumeyrolle S., D'Anna B., Decesari S., Gilardoni S., Fischer M., Fjaeraa A. M., Fountoukis C., George C., Gomes L., Halloran P., Hamburger T., Harrison R. M., Herrmann H., Hoffmann T., Hoose C., Hu M., Hyvärinen A., Hõrrak U., Iinuma Y., Iversen T., Josipovic M., Kanakidou M., Kiendler-Scharr A., Kirkevåg A., Kiss G., Klimont Z., Kolmonen P., Komppula M., Kristjánsson J.-E., Laakso L., Laaksonen A., Labonnote L., Lanz V. A., Lehtinen K. E. J., Rizzo L. V., Makkonen R., Manninen H. E., McMeeking G., Merikanto J., Minikin A., Mirme S., Morgan W. T., Nemitz E., O'Donnell D., Panwar T. S., Pawlowska H., Petzold A., Pienaar J. J., Pio C., Plass-Duelmer C., Prévôt A. S. H., Pryor S., Reddington C. L., Roberts G., Rosenfeld D., Schwarz J., Seland Ø., Sellegri K., Shen X. J., Shiraiwa M., Siebert H., Sierau B., Simpson D., Sun J. Y., Topping D., Tunved P., Vaattovaara P., Vakkari V., Veefkind J. P., Visschedijk A., Vuollekoski H., Vuolo R., Wehner B., Wildt J., Woodward S., Worsnop D. R., van Zadelhoff G.-J., Zardini A. A., Zhang K., van Zyl P. G., Kerminen V.-M., Carslaw K. S., Pandis S. N. (2011). General overview: European Integrated project on Aerosol Cloud Climate and Air Quality interactions (EUCAARI) integrating aerosol research from nano to global scales. Atmos. Chem. Phys..

[cit54] Williams J., Crowley J., Fischer H., Harder H., Martinez M., Petäjä T., Rinne J., Bäck J., Boy M., Dal Maso M., Hakala J., Kajos M., Keronen P., Rantala P., Aalto J., Aaltonen H., Paatero J., Vesala T., Hakola H., Levula J., Pohja T., Herrmann F., Auld J., Mesarchaki E., Song W., Yassaa N., Nölscher A., Johnson A. M., Custer T., Sinha V., Thieser J., Pouvesle N., Taraborrelli D., Tang M. J., Bozem H., Hosaynali-Beygi Z., Axinte R., Oswald R., Novelli A., Kubistin D., Hens K., Javed U., Trawny K., Breitenberger C., Hidalgo P. J., Ebben C. J., Geiger F. M., Corrigan A. L., Russell L. M., Ouwersloot H. G., Vilà-Guerau de Arellano J., Ganzeveld L., Vogel A., Beck M., Bayerle A., Kampf C. J., Bertelmann M., Köllner F., Hoffmann T., Valverde J., González D., Riekkola M.-L., Kulmala M., Lelieveld J. (2011). The summertime Boreal forest field measurement intensive (HUMPPA-COPEC-2010): an overview of meteorological and chemical influences. Atmos. Chem. Phys..

[cit55] Boy M., Mogensen D., Smolander S., Zhou L., Nieminen T., Paasonen P., Plass-Dülmer C., Sipilä M., Petäjä T., Mauldin L., Berresheim H., Kulmala M. (2013). Oxidation of SO_2_ by stabilized Criegee intermediate (sCI) radicals as a crucial source for atmospheric sulfuric acid concentrations. Atmos. Chem. Phys..

[cit56] Liebmann J., Karu E., Sobanski N., Schuladen J., Ehn M., Schallhart S., Quéléver L., Hellen H., Hakola H., Hoffmann T., Williams J., Fischer H., Lelieveld J., Crowley J. N. (2018). Direct measurement of NO_3_ radical reactivity in a boreal forest. Atmos. Chem. Phys..

[cit57] Peräkylä O., Vogt M., Tikkanen O.-P., Laurila T., Kajos M. K., Rantala P. A., Patokoski J., Aalto J., Yli-Juuti T., Ehn M., Sipilä M., Paasonen P., Rissanen M., Nieminen T., Taipale R., Keronen P., Lappalainen H. K., Ruuskanen T. M., Rinne J., Kerminen V.-M., Kulmala M., Bäck J., Petäjä T. (2014). Monoterpenes' oxidation capacity and rate over a boreal forest: temporal variation and connection to growth of newly formed particles. Boreal Environ. Res..

[cit58] Kontkanen J., Paasonen P., Aalto J., Bäck J., Rantala P., Petäjä T., Kulmala M. (2016). Simple proxies for estimating the concentrations of monoterpenes and their oxidation products at a boreal forest site. Atmos. Chem. Phys..

[cit59] Hari P., Kulmala M. (2005). Station for Measuring Ecosystem-Atmosphere Relations (SMEAR II). Boreal Environ. Res..

[cit60] Bäck J., Aalto J., Henriksson M., Hakola H., He Q., Boy M. (2012). Chemodiversity of a Scots pine stand and implications for terpene air concentrations. Biogeosciences.

[cit61] Hellén H., Praplan A. P., Tykkä T., Ylivinkka I., Vakkari V., Bäck J., Petäjä T., Kulmala M., Hakola H. (2018). Long-term measurements of volatile organic compounds highlight the importance of sesquiterpenes for the atmospheric chemistry of a boreal forest. Atmos. Chem. Phys..

[cit62] Smolander S., He Q., Mogensen D., Zhou L., Bäck J., Ruuskanen T., Noe S., Guenther A., Aaltonen H., Kulmala M., Boy M. (2014). Comparing three vegetation monoterpene emission models to measured gas concentrations with a model of meteorology, air chemistry and chemical transport. Biogeosciences.

[cit63] Zhou P., Ganzeveld L., Rannik Ü., Zhou L., Gierens R., Taipale D., Mammarella I., Boy M. (2017). Simulating ozone dry deposition at a boreal forest with a multi-layer canopy deposition model. Atmos. Chem. Phys..

[cit64] Zhou P., Ganzeveld L., Taipale D., Rannik Ü., Rantala P., Rissanen M. P., Chen D., Boy M. (2017). Boreal forest BVOC exchange: emissions *versus* in-canopy sinks. Atmos. Chem. Phys..

[cit65] Zhou L., Nieminen T., Mogensen D., Smolander S., Rusanen A., Kulmala M., Boy M. (2014). SOSAA—a new model to simulate the concentrations of organic vapours, sulphuric acid and aerosols inside the ABL—Part 2: aerosol dynamics and one case study at a boreal forest site. Boreal Environ. Res..

[cit66] Sogachev A., Menzhulin G. V., Heimann M., Lloyd J. (2002). A simple three-dimensional canopy – planetary boundary layer simulation model for scalar concentrations and fluxes. Tellus B.

[cit67] Sogachev A., Panferov O., Gravenhorst G., Vesala T. (2005). Numerical analysis of flux footprints for different landscapes. Theor. Appl. Climatol..

[cit68] Sogachev A., Panferov O. (2006). Modification of Two-Equation Models to Account for Plant Drag. Boundary-Layer Meteorology.

[cit69] Damian V., Sandu A., Damian M., Potra F., Carmichael G. R. (2002). The kinetic preprocessor KPP-a software environment for solving chemical kinetics. Comput. Chem. Eng..

[cit70] Jenkin M. E., Saunders S. M., Pilling M. J. (1997). The tropospheric degradation of volatile organic compounds: a protocol for mechanism development. Atmos. Environ..

[cit71] Saunders S. M., Jenkin M. E., Derwent R. G., Pilling M. J. (2003). Protocol for the development of the Master Chemical Mechanism, MCM v3 (Part A): tropospheric degradation of non-aromatic volatile organic compounds. Atmos. Chem. Phys..

[cit72] Jenkin M. E., Wyche K. P., Evans C. J., Carr T., Monks P. S., Alfarra M. R., Barley M. H., McFiggans G. B., Young J. C., Rickard A. R. (2012). Development and chamber evaluation of the MCM v3.2 degradation scheme for β-caryophyllene. Atmos. Chem. Phys..

[cit73] Ganzeveld L. N., Lelieveld J., Dentener F. J., Krol M. C., Roelofs G. J. (2002). Atmosphere-biosphere trace gas exchanges simulated with a single-column model. J. Geophys. Res.: Atmos..

[cit74] Dee D. P., Uppala S. M., Simmons A. J., Berrisford P., Poli P., Kobayashi S., Andrae U., Balmaseda M. A., Balsamo G., Bauer P., Bechtold P., Beljaars A. C. M., van de Berg L., Bidlot J., Bormann N., Delsol C., Dragani R., Fuentes M., Geer A. J., Haimberger L., Healy S. B., Hersbach H., Hólm E. V., Isaksen L., Kållberg P., Köhler M., Matricardi M., McNally A. P., Monge-Sanz B. M., Morcrette J.-J., Park B.-K., Peubey C., de Rosnay P., Tavolato C., Thépaut J.-N., Vitart F. (2011). The era-interim reanalysis: configuration and performance of the data assimilation system. Q. J. R. Metereol. Soc..

[cit75] Roldin P., Swietlicki E., Massling A., Kristensson A., Löndahl J., Eriksson A., Pagels J., Gustafsson S. (2011). Aerosol ageing in an urban plume – implication for climate. Atmos. Chem. Phys..

[cit76] Toon O. B., McKay C. P., Ackerman T. P., Santhanam K. (1989). Rapid calculation of radiative heating rates and photodissociation rates in inhomogeneous multiple scattering atmospheres. J. Geophys. Res., D: Atmos..

[cit77] Mauldin III R. L., Berndt T., Sipilä M., Paasonen P., Petäjä T., Kim S., Kurtén T., Stratmann F., Kerminen V.-M., Kulmala M. (2012). A new atmospherically relevant oxidant. Nature.

[cit78] Welz O., Savee J. D., Osborn D. L., Vasu S. S., Percival C. J., Shallcross D. E., Taatjes C. A. (2012). Direct kinetic measure-ments of Criegee Intermediate (CH2OO) formed by reaction of CH2I with O2. Science.

[cit79] Cohen M. A., Ryan P. B. (1989). Observations Less than the Analytical Limit of Detection: A New Approach. JAPCA.

[cit80] Pirjola L., Kulmala M., Bischoff A., Wilck M., Stratmann F. (1998). Effects of aerosol dynamics on the formation of sulphuric acid aerosols. J. Aerosol Sci..

[cit81] Laakso L., Kulmala M., Lehtinen K. E. J. (2003). Effect of condensation rate enhancement factor on 3-nm (diameter) particle formation in binary ion-induced and homogeneous nucleation. J. Geophys. Res., D: Atmos..

[cit82] Wilks D. S. (1997). Resampling Hypothesis Tests for Autocorrelated Fields. J. Clim..

[cit83] Asmi A., Collaud Coen M., Ogren J. A., Andrews E., Sheridan P., Jefferson A., Weingartner E., Baltensperger U., Bukowiecki N., Lihavainen H., Kivekäs N., Asmi E., Aalto P. P., Kulmala M., Wiedensohler A., Birmili W., Hamed A., O'Dowd C., Jennings S. G., Weller R., Flentje H., Fjaeraa A. M., Fiebig M., Myhre C. L., Hallar A. G., Swietlicki E., Kristensson A., Laj P. (2013). Aerosol decadal trends – Part 2: *in situ* aerosol particle number concentrations at GAW and ACTRIS stations. Atmos. Chem. Phys..

[cit84] Hipel K. W. and McLeod A. I., Time Series Modelling of Water Resources and Environmental Systems, Elsevier, Amsterdam, 1994, vol. 45

[cit85] Hussain M., Mahmud I. (2019). pyMannKendall: a python package for non parametric Mann Kendall family of trend tests. J. Open Source Softw..

[cit86] Ma Z., Xu J., Quan W., Zhang Z., Lin W., Xu X. (2016). Significant increase of surface ozone at a rural site, north of eastern China. Atmos. Chem. Phys..

[cit87] Atkinson R., Aschmann S. M., Arey J., Shorees B. (1992). Formation of OH radicals in the gas phase reactions of O_3_ with a series of terpenes. J. Geophys. Res.: Atmos..

[cit88] Guenther A. B., Jiang X., Heald C. L., Sakulyanontvittaya T., Duhl T., Emmons L. K., Wang X. (2012). The Model of Emissions of Gases and Aerosols from Nature version 2.1 (MEGAN2.1): an extended and updated framework for modeling biogenic emissions. Geosci. Model Dev..

[cit89] Foken T. (2008). The energy balance closure problem: an overview. Ecol. Appl..

[cit90] Nieminen T., Asmi A., Dal Maso M., Aalto P. P., Keronen P., Petäjä T., Kulmala M., Kerminen V.-M. (2014). Trends in atmospheric new-particle formation: 16 years of observations in a boreal-forest environment. Boreal Environ. Res..

[cit91] Seinfeld J. H. and Pandis S. N., Atmospheric Chemistry and Physics: From Air Pollution to Climate Change, John Wiley & Sons, New York, 2nd edn, 2006

[cit92] Hoesly R. M., Smith S. J., Feng L., Klimont Z., Janssens-Maenhout G., Pitkanen T., Seibert J. J., Vu L., Andres R. J., Bolt R. M., Bond T. C., Dawidowski L., Kholod N., Kurokawa J.-I., Li M., Liu L., Lu Z., Moura M. C. P., O'Rourke P. R., Zhang Q. (2018). Historical (1750–2014) anthropogenic emissions of reactive gases and aerosols from the Community Emissions Data System (CEDS). Geosci. Model Dev..

[cit93] Mäki M., Heinonsalo J., Hellén H., Bäck J. (2017). Contribution of understorey vegetation and soil processes to boreal forest isoprenoid exchange. Biogeosciences.

[cit94] Mäki M., Aalto J., Hellén H., Pihlatie M., Bäck J. (2019). Interannual and Seasonal Dynamics of Volatile Organic Compound Fluxes From the Boreal Forest Floor. Front. Plant Sci..

[cit95] Aaltonen H., Pumpanen J., Pihlatie M., Hakola H., Hellén H., Kulmala L., Vesala T., Bäck J. (2011). Boreal pine forest floor biogenic volatile organic compound emissions peak in early summer and autumn. Agricultural and Forest Meteorology.

[cit96] Hens K., Novelli A., Martinez M., Auld J., Axinte R., Bohn B., Fischer H., Keronen P., Kubistin D., Nölscher A. C., Oswald R., Paasonen P., Petäjä T., Regelin E., Sander R., Sinha V., Sipilä M., Taraborrelli D., Tatum Ernest C., Williams J., Lelieveld J., Harder H. (2014). Observation and modelling of HO_*x*_ radicals in a boreal forest. Atmos. Chem. Phys..

[cit97] Boy M., Hellmuth O., Korhonen H., Nilsson E. D., ReVelle D., Turnipseed A., Arnold F., Kulmala M. (2006). MALTE-model to predict new aerosol formation in the lower troposphere. Atmos. Chem. Phys..

[cit98] Petrenko V. V., Martinerie P., Novelli P., Etheridge D. M., Levin I., Wang Z., Blunier T., Chappellaz J., Kaiser J., Lang P., Steele L. P., Hammer S., Mak J., Langenfelds R. L., Schwander J., Severinghaus J. P., Witrant E., Petron G., Battle M. O., Forster G., Sturges W. T., Lamarque J.-F., Steffen K., White J. W. C. (2013). A 60 year record of atmospheric carbon monoxide reconstructed from greenland firn air. Atmos. Chem. Phys..

[cit99] Jiang Z., Worden J. R., Worden H., Deeter M., Jones D. B. A., Arellano A. F., Henze D. K. (2017). A 15-year record of CO emissions constrained by MOPITT CO observations. Atmos. Chem. Phys..

[cit100] Zhang D., Zhang R. (2005). Ozonolysis of α-pinene and β-pinene: kinetics and mechanism. J. Chem. Phys..

[cit101] Nguyen T. L., Peeters J., Vereecken L. (2009). Theoretical study of the gas-phase ozonolysis of β-pinene (C10H16). Phys. Chem. Chem. Phys..

[cit102] Alam M. S., Rickard A. R., Camredon M., Wyche K. P., Carr T., Hornsby K. E., Monks P. S., Bloss W. J. (2013). Radical Product Yields from the Ozonolysis of Short Chain Alkenes under Atmospheric Boundary Layer Conditions. J. Phys. Chem. A.

[cit103] Rantala P., Aalto J., Taipale R., Ruuskanen T. M., Rinne J. (2015). Annual cycle of volatile organic compound exchange between a boreal pine forest and the atmosphere. Biogeosciences.

[cit104] Sporre M. K., Blichner S. M., Karset I. H. H., Makkonen R., Berntsen T. K. (2019). BVOC–aerosol–climate feedbacks investigated using NorESM. Atmos. Chem. Phys..

[cit105] Conrad R. (1996). Soil microorganisms as controllers of atmospheric trace gases (H_2_, CO, CH_4_, OCS, N_2_O, and NO). Microbiol. Rev..

[cit106] Kesik M., Ambus P., Baritz R., Brüggemann N., Butterbach-Bahl K., Damm M., Duyzer J., Horváth L., Kiese R., Kitzler B., Leip A., Li C., Pihlatie M., Pilegaard K., Seufert S., Simpson D., Skiba U., Smiatek G., Vesala T., Zechmeister-Boltenstern S. (2005). Inventories of N_2_O and NO emissions from European forest soils. Biogeosciences.

[cit107] Jokinen T., Sipilä M., Junninen H., Ehn M., Lönn G., Hakala J., Petäjä T., Mauldin III R. L., Kulmala M., Worsnop D. R. (2012). Atmospheric sulphuric acid and neutral cluster measurements using CI-APi-TOF. Atmos. Chem. Phys..

[cit108] Dada L., Ylivinkka I., Baalbaki R., Li C., Guo Y., Yan C., Yao L., Sarnela N., Jokinen T., Daellenbach K. R., Yin R., Deng C., Chu B., Nieminen T., Wang Y., Lin Z., Thakur R. C., Kontkanen J., Stolzenburg D., Sipilä M., Hussein T., Paasonen P., Bianchi F., Salma I., Weidinger T., Pikridas M., Sciare J., Jiang J., Liu Y., Petäjä T., Kerminen V.-M., Kulmala M. (2020). Sources and sinks driving sulfuric acid concentrations in contrasting environments: implications on proxy calculations. Atmos. Chem. Phys..

[cit109] Atkinson R., Arey J. (2003). Atmospheric Degradation of Volatile Organic Compounds. Chem. Rev..

[cit110] Fry J. L., Draper D. C., Barsanti K. C., Smith J. N., Ortega J., Winkler P. M., Lawler M. J., Brown S. S., Edwards P. M., Cohen R. C., Lee L. (2014). Secondary Organic Aerosol Formation and Organic Nitrate Yield from NO_3_ Oxidation of Biogenic Hydrocarbons. Environ. Sci. Technol..

[cit111] Makkonen R., Asmi A., Kerminen V.-M., Boy M., Arneth A., Hari P., Kulmala M. (2012). Air pollution control and decreasing new particle formation lead to strong climate warming. Atmos. Chem. Phys..

