# Supplementary material for: A modelling study of OH, NO3 and H2SO4 in 2007–2018 at SMEAR II, Finland: analysis of long-term trends
Source: Environ Sci Atmos. 2021 Aug 4;1(6):449–72. doi: 10.1039/d1ea00020a (PMC8459646; doi:10.1039/d1ea00020a)
Supplement: EA-001-D1EA00020A-s001 [file EA-001-D1EA00020A-s001.pdf]

Electronic Supplementary Material (ESI) for Environmental Science: Atmospheres.
This journal is © The Royal Society of Chemistry 2021

Supplementary material for

Modelling study of OH, NO_3 and H_2SO_4 in 2007 - 2018 at SMEAR II, Finland: analysis of long-term trends

Dean Chen, Carlton Xavier, Petri Clusius, Tuomo Nieminen, Pontus Roldin, Ximeng Qi, Lukas Pichelstorfer, Markku Kulmala, Pekka Rantala, Juho Aalto, Nina Sarnela, Pasi Kolari, Petri Keronen, Matti P. Rissanen, Ditte Taipale, Benjamin Foreback, Metin Baykara, Putian Zhou, Michael Boy

SO_2	90th	75th	25th	10th	Median	Mean
**2007**	10.41	24.93	68.77	78.90	50.41	0.00
**2008**	6.85	15.89	65.21	78.08	39.45	0.00
**2009**	6.03	21.64	63.01	75.62	43.56	0.00
**2010**	2.19	8.77	37.26	49.59	20.82	0.00
**2011**	7.67	17.81	44.38	53.97	32.88	0.00
**2012**	13.97	24.38	55.07	69.32	39.73	0.00
**2013**	12.05	24.66	60.27	72.05	43.56	0.00
**2014**	19.45	37.26	73.42	81.92	58.36	0.00
**2015**	22.19	35.89	68.49	79.73	53.70	0.00
**2016**	34.52	55.62	88.77	91.51	79.18	0.00
**2017**	48.49	68.77	95.89	98.36	88.22	0.00
**2018**	27.40	45.75	77.53	83.29	62.47	0.00
**NO**	**90th**	**75th**	**25th**	**10th**	**Median**	**Mean**
**2007**	44.38	72.60	100.00	100.00	99.45	67.12
**2008**	41.37	72.33	100.00	100.00	98.63	70.41
**2009**	48.49	82.74	100.00	100.00	99.73	72.05
**2010**	35.62	68.49	100.00	100.00	98.08	62.47
**2011**	31.78	61.37	100.00	100.00	96.71	59.18
**2012**	41.64	73.70	100.00	100.00	98.63	67.67
**2013**	53.70	78.08	100.00	100.00	100.00	75.07
**2014**	46.03	77.53	100.00	100.00	99.18	72.88
**2015**	44.66	76.99	99.73	100.00	99.73	75.62
**2016**	33.97	64.66	99.73	100.00	94.52	63.01
**2017**	49.04	80.00	99.73	100.00	98.36	77.81
**2018**	35.89	67.67	99.73	100.00	98.36	63.56

Table S1: The percentages of the 90th, 75th, 25th, 10th percentiles, median and mean values of daily data under the limit of detection at smear II for SO2 and NO for years 2007-2018.

		Method: RLM			Method: MK		Signi-ficance
Paramter	Time	Trend / year	90% confidence interval		Trend / year	P-value	
SO_2 concentration	Daily	-5.4281	-8.0980	-3.6522	-3.9111	0.0129	decreasing
	Day	-4.9980	-7.6658	-3.2234	-2.6538	0.0185	decreasing
	Night	-5.3471	-8.0738	-3.5505	-3.0735	0.0130	decreasing
O_3 concentration	Daily	-0.1131	-0.8641	+0.0485	+0.1609	0.6121	no trend
	Day	-0.1795	-0.8778	-0.0126	+0.0348	0.8840	no trend
	Night	+0.0436	-0.7623	+0.2100	+0.2958	0.3301	no trend
CO concentration	Daily	-0.4629	-1.6208	+0.3779	-0.4166	0.4725	no trend
	Day	-0.4748	-1.6337	+0.3374	-0.4401	0.4556	no trend
	Night	-0.4343	-1.6262	+0.4226	-0.4091	0.4990	no trend
NO concentration	Daily	-0.3698	-1.1336	+0.0814	-0.0198	0.9072	no trend
	Day	-0.9064	-2.1235	-0.1924	-0.1267	0.6312	no trend
	Night	+0.0010	-0.0218	+0.0230	+0.0001	0.9804	no trend
NO_2 concentration	Daily	-3.8029	-5.9864	-1.8669	-3.8871	0.0307	decreasing
	Day	-3.3308	-5.5153	-1.4325	-3.3120	0.0451	decreasing
	Night	-4.1751	-6.2646	-2.3209	-4.2051	0.0193	decreasing
Global short wave radiation	Daily	+0.2381	-0.8528	+0.6783	+0.2735	0.1104	no trend
	Day	+0.2583	-1.5394	+1.0469	+0.4675	0.1768	no trend
	Night	+0.0099	-0.0026	+0.0253	+0.0077	0.3529	no trend
Temperature	Daily	+0.0772	+0.0242	+0.2369	+0.0292	0.6322	no trend
	Day	+0.0778	+0.0244	+0.2358	+0.0337	0.5686	no trend
	Night	+0.0777	+0.0300	+0.2347	+0.0230	0.7141	no trend
Absolute humidity	Daily	-0.1050	-0.4659	+0.6998	-0.2723	0.3430	no trend
	Day	-0.0708	-0.4430	+0.7587	-0.2370	0.4172	no trend
	Night	-0.1662	-0.5497	+0.6451	-0.3300	0.2866	no trend
Condensation sink	Daily	-1.5859	-3.4925	-0.1168	-1.0829	0.3562	no trend
	Day	-1.6548	-3.5853	-0.1989	-1.1005	0.3339	no trend
	Night	-1.3936	-3.2220	-0.0211	-0.8945	0.4192	no trend

Table S2: Yearly trends calculated by two different statistical methods (RLM = robust linear method, MK = Mann Kendall), 90% confidence interval (first and second numbers show the 5th and 95th percentiles of the yearly trend slopes obtained from 10 000 bootstrapping iterations, respectively) and P_{MK} values of different measured parameters. The first, second and third rows for each parameter represent daily, daytime and nighttime values, respectively. All data represent the mean values from the height levels 4.2, 8.4, 16.8, 33.6, 50.4 and 67.2 m. Detailed statistical methods are explained in Section 2.3.

Season	Daily			Day			Night		
	Trend / year	90% conficence interval		Trend / year	90% conficence interval		Trend / year	90% conficence interval	
	**SO_2 concentration**								
Winter	-10.177	-16.990	-4.851	-10.005	-17.069	-4.077	-9.972	-16.707	-4.732
Spring	-4.095	-7.728	-0.525	-3.204	-6.811	+0.264	-4.536	-8.293	-0.858
Summer	-4.567	-7.747	-1.175	-4.479	-7.881	-0.875	-3.905	-7.134	-0.585
Autumn	-3.594	-5.175	-1.978	-2.757	-4.032	-1.473	-3.537	-5.043	-1.940
	**O_3 concentration**								
Winter	+0.743	-0.491	1.450	+0.886	-0.313	+1.514	+0.668	-0.585	+1.379
Spring	+0.131	-0.509	+0.823	+0.085	-0.515	+0.751	+0.238	-0.452	+0.948
Summer	-0.278	-1.117	+0.476	-0.372	-1.160	+0.346	-0.017	-0.892	+0.790
Autumn	+0.037	-0.931	+0.979	-0.271	-1.180	+0.601	+0.239	-0.753	+1.267
	**CO concentration**								
Winter	-0.894	-1.958	-0.283	-0.981	-2.009	-0.394	-0.848	-1.933	-0.242
Spring	-0.962	-1.721	-0.322	-0.963	-1.708	-0.310	-0.968	-1.735	-0.297
Summer	-0.543	-2.497	+1.414	-0.540	-2.473	+1.431	-0.525	-2.407	+1.452
Autumn	+0.750	-0.891	+2.443	+0.788	-0.807	+2.454	+0.779	-0.787	+2.482
	**NO concentration**								
Winter	-2.608	-4.963	-1.665	-5.929	-10.153	-3.856	-0.223	-0.547	+0.102
Spring	+0.626	-0.838	+1.948	+0.801	-1.183	+2.558	+0.012	-0.007	+0.029
Summer	-0.021	-0.499	+0.488	-0.002	-0.609	+0.675	-0.002	-0.009	+0.004
Autumn	-0.269	-0.803	+0.197	-0.646	-1.610	+0.307	+0.015	-0.028	+0.059
	**NO_2 concentration**								
Winter	-8.486	-12.486	-4.874	-8.610	-12.519	-5.049	-8.206	-12.464	-4.527
Spring	-2.317	-6.072	+0.991	-1.475	-5.211	+1.994	-3.082	-6.180	-0.215
Summer	-1.336	-4.357	+1.923	-0.712	-3.657	+2.448	-2.001	-4.947	+1.038
Autumn	-4.132	-5.859	-1.956	-3.672	-5.284	-1.533	-4.644	-6.659	-2.247
	**Global short wave rediation**								
Winter	-0.199	-0.680	-0.217	-0.740	-2.046	-0.801	+0.030	+0.004	+0.066
Spring	+1.370	-0.016	+3.430	+2.421	-0.013	+5.518	-0.009	-0.017	+0.002
Summer	+0.851	-1.249	+2.611	+0.902	-1.740	+3.335	-0.002	-0.060	+0.052
Autumn	+0.023	-0.635	+0.303	-0.057	-1.397	+0.626	-0.001	-0.014	+0.013
	**Temperature**								
Winter	+0.234	-0.043	+0.676	+0.228	-0.063	+0.670	+0.237	-0.038	+0.685
Spring	-0.024	-0.102	+0.125	-0.021	-0.100	+0.127	-0.025	-0.110	+0.118
Summer	-0.022	-0.169	+0.135	-0.022	-0.177	+0.141	-0.004	-0.143	+0.141
Autumn	-0.026	-0.140	+0.139	-0.025	-0.139	+0.131	-0.024	-0.138	+0.142
	**Absolute humidity**								
Winter	+0.210	-1.233	+2.857	+0.195	-1.219	+2.879	0.147	-1.295	+2.734
Spring	-0.191	-0.635	+0.505	-0.131	-0.580	+0.591	-0.293	-0.756	+0.399
Summer	-0.260	-0.670	+0.181	-0.264	-0.692	+0.187	-0.290	-0.691	+0.142
Autumn	-0.371	-1.018	+0.105	-0.371	-1.042	+0.111	-0.361	-0.981	+0.104
	**Condensation sink**								
Winter	-4.761	-7.839	-2.952	-5.188	-8.585	-3.362	-4.336	-7.408	-2.636
Spring	-1.107	-3.509	+1.505	-1.215	-3.726	+1.510	-1.144	-3.423	+1.359
Summer	-0.042	-1.636	+1.518	-0.134	-1.730	+1.480	+0.253	-1.319	+1.852
Autumn	-0.872	-3.061	+1.071	-0.676	-3.009	+1.450	-0.807	-2.982	+1.142

Table S3: Seasonal yearly trends for same parameters as in table S1 calculated by RLM method with 90% confidence interval (first and second numbers show the 5th and 95th percentiles of the yearly trend slopes obtained from 10 000 bootstrapping iterations, respectively). The first, second and third set of columns for each parameter represent daily, daytime and nighttime values, respectively. All data represent the mean values from the height levels 4.2, 8.4, 16.8, 33.6, 50.4 and 67.2 m. Detailed statistical methods are explained in Section 2.3.

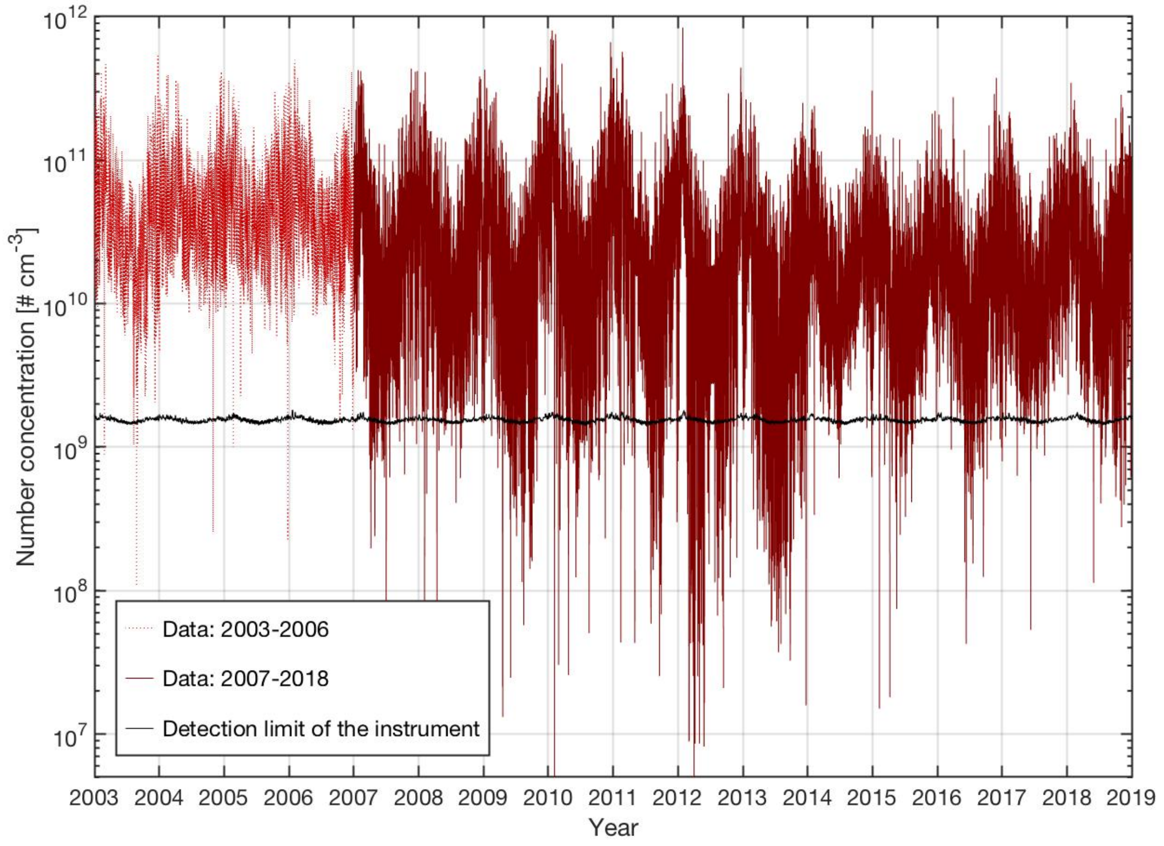

Figure S1: NO_2 measurements from 2003-2018 at the SMEAR II

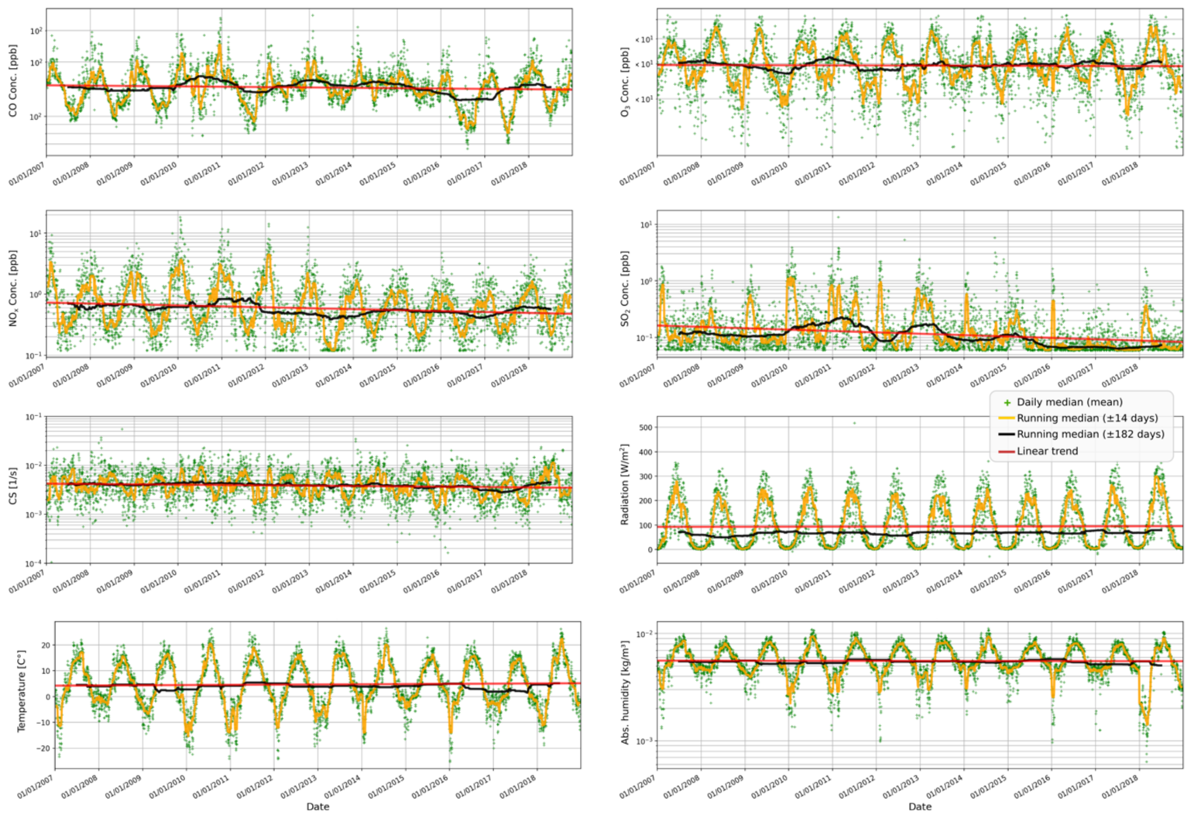

Figure S2: Data and trend of selected measured parameters from SMEAR II for the years 2007-2018 (CS = condensation sink; Radiation = short wave irradiance).

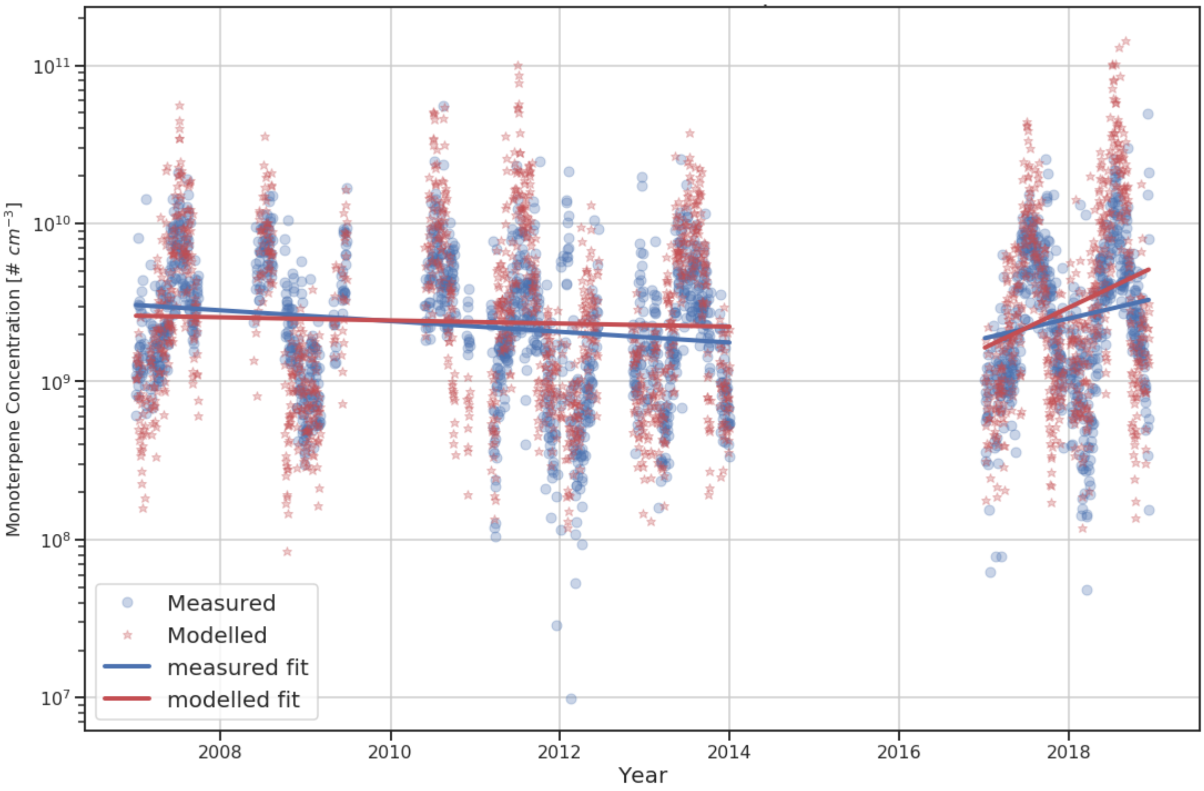

Figure S3: Time series of modelled (red star) and measured (blue circle) monoterpene concentrations from 2007 to 2018. The linear fits of the logarithmic data are plotted as solid lines in the same colour for modelled and measured data. The modelled data points are plotted only when measured data are available.

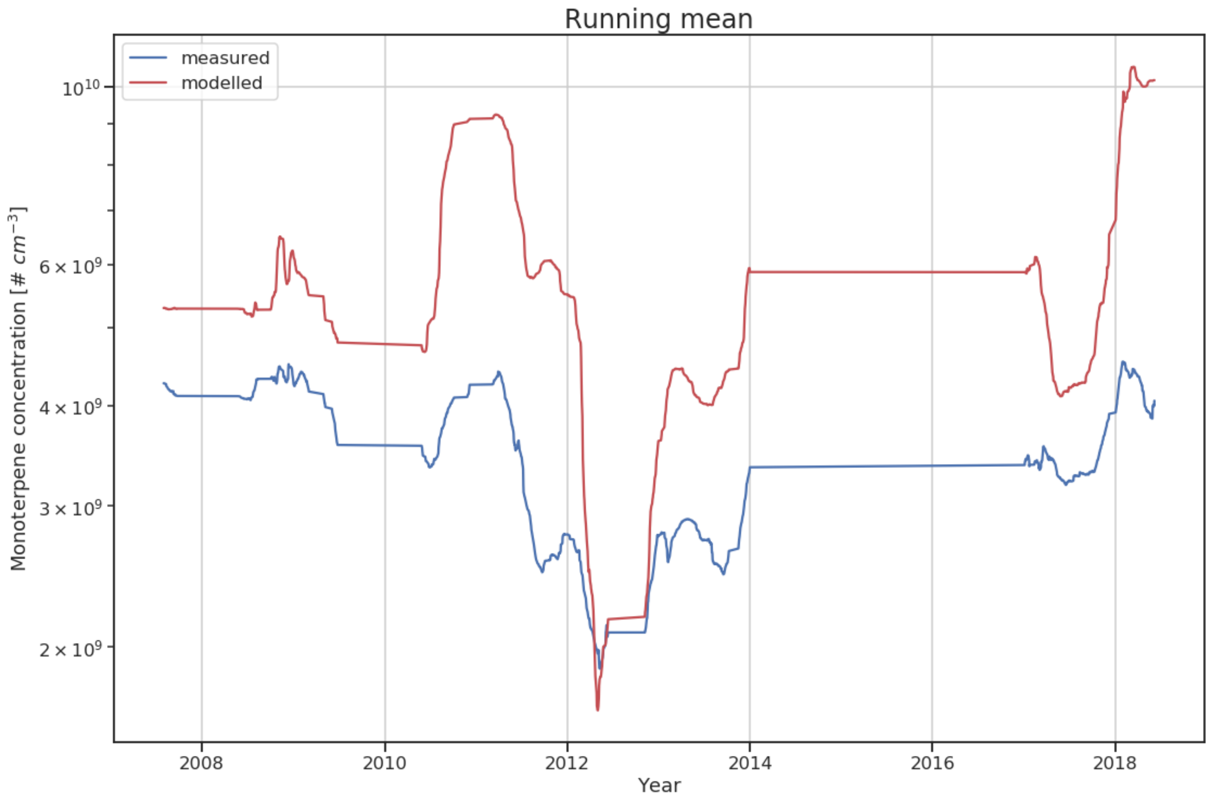

Figure S4: One-year running mean of modelled (red) and measured (blue) monoterpene concentrations from 2007 to 2018.

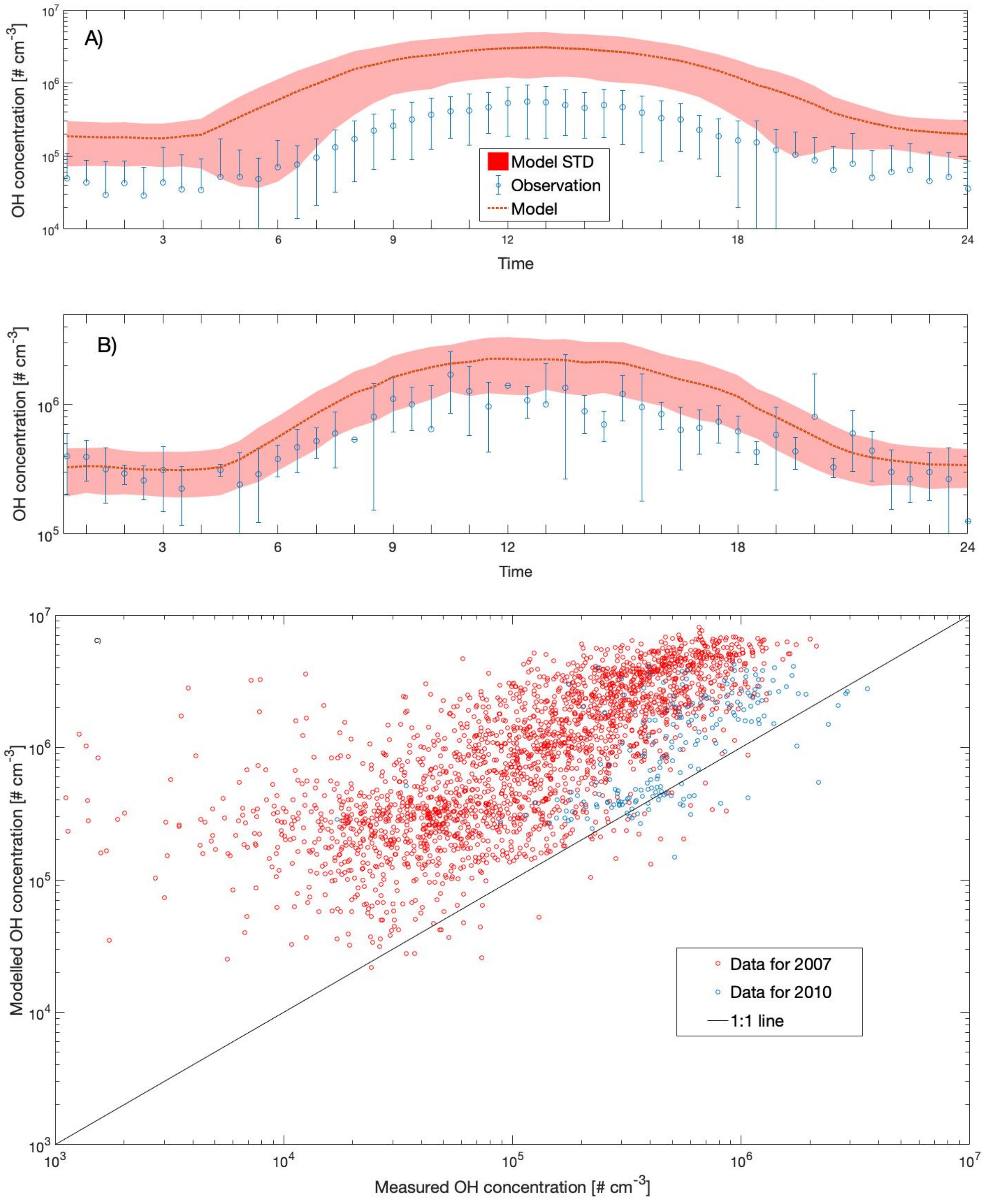

Figure S5: Diurnal cycle of measured and modelled OH concentrations for two campaigns in 2007 and 2010, respectively (A and B); scatter plot for all data points during the two campaigns (C).

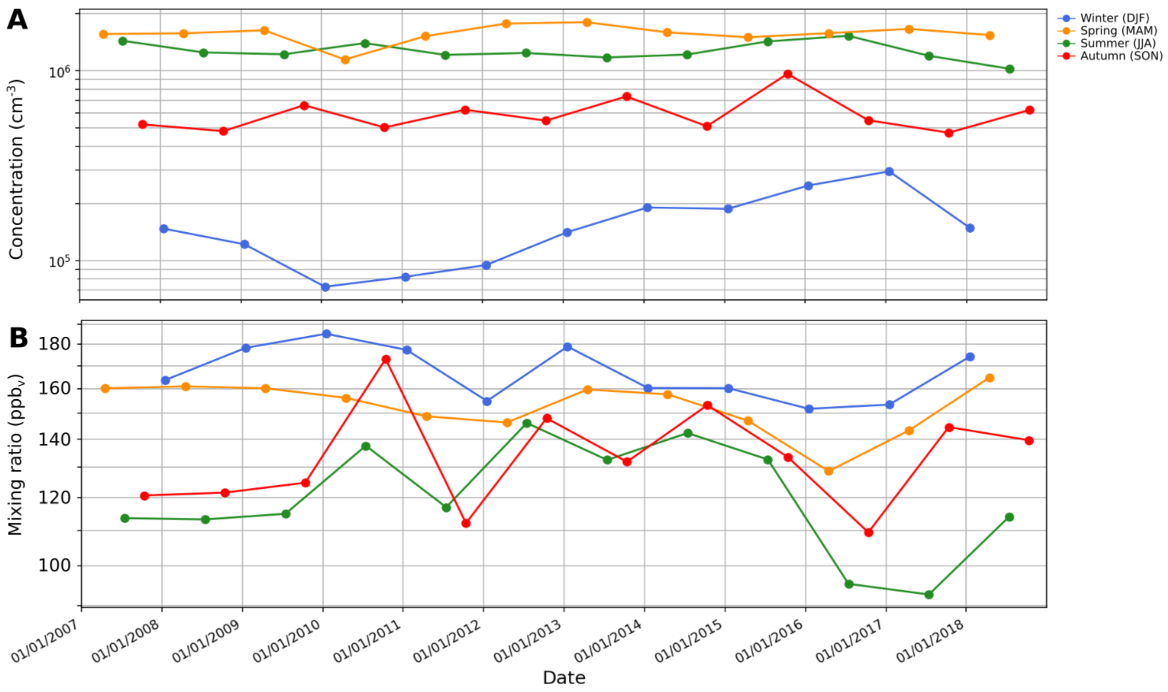

Figure S6: Seasonal medians of daytime OH (A) and CO (B) concentrations in logarithm scale for winter (blue), spring (orange), summer (green) and autumn (red) from 2007 to 2018.

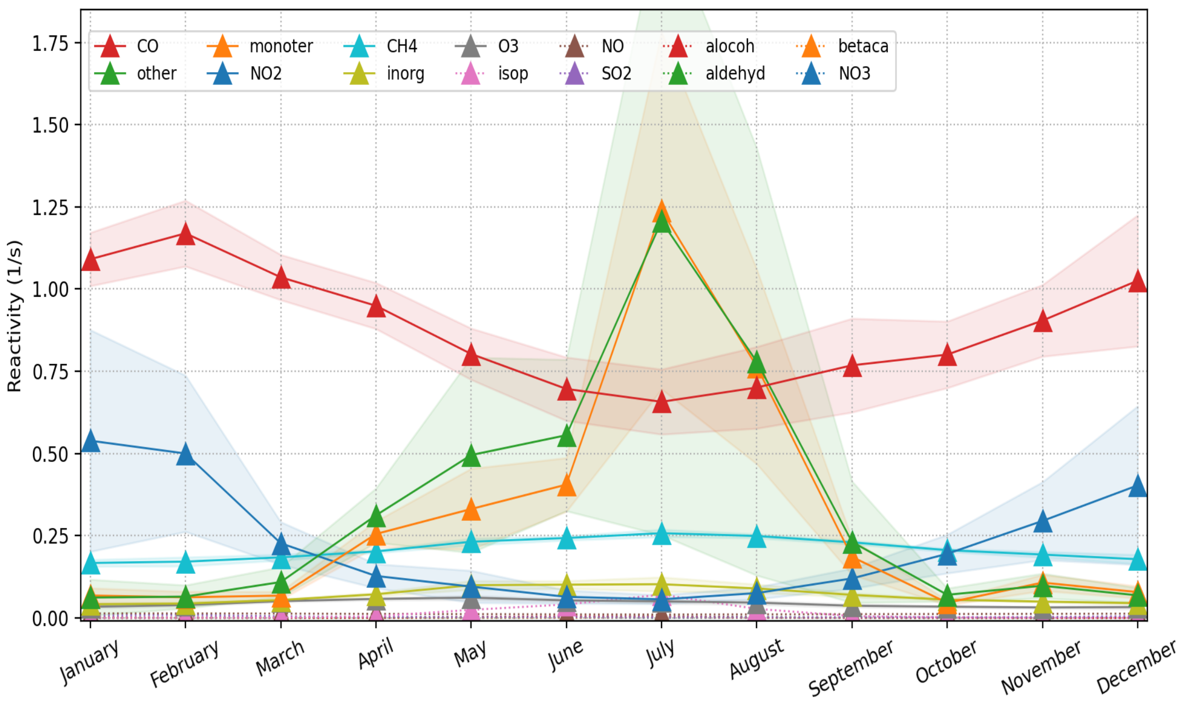

Figure S7: Mean monthly reactivity in 2007–2018 (and +/- 1 standard deviation for most significant compounds/classes)

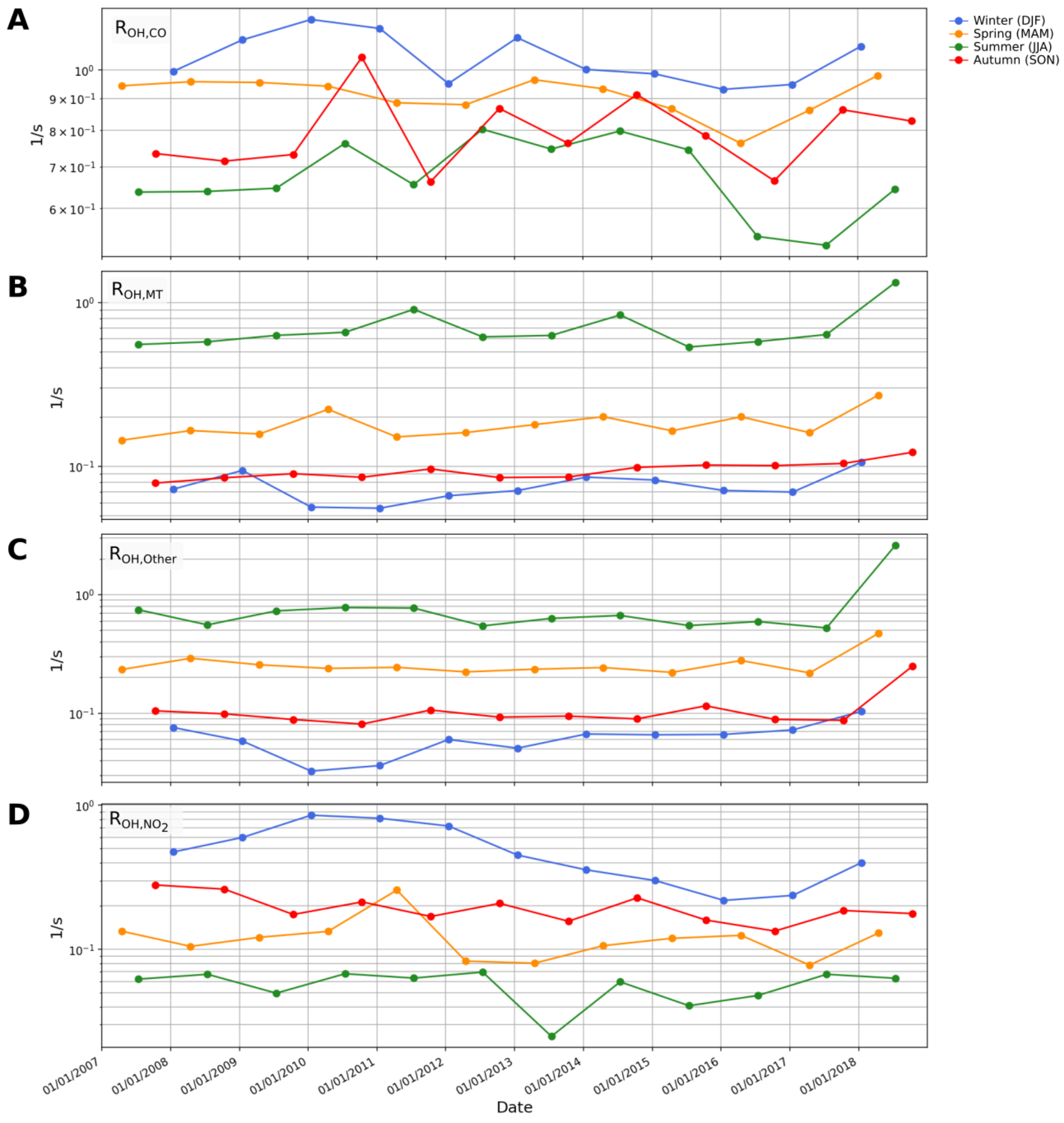

Figure S8: Seasonal medians of the four major daytime OH-reactivity terms for winter (blue), spring (orange), summer (green) and autumn (red) from 2007 to 2018: CO (A), monoterpenes (B), higher order reactions (C) and NO_2 (D).

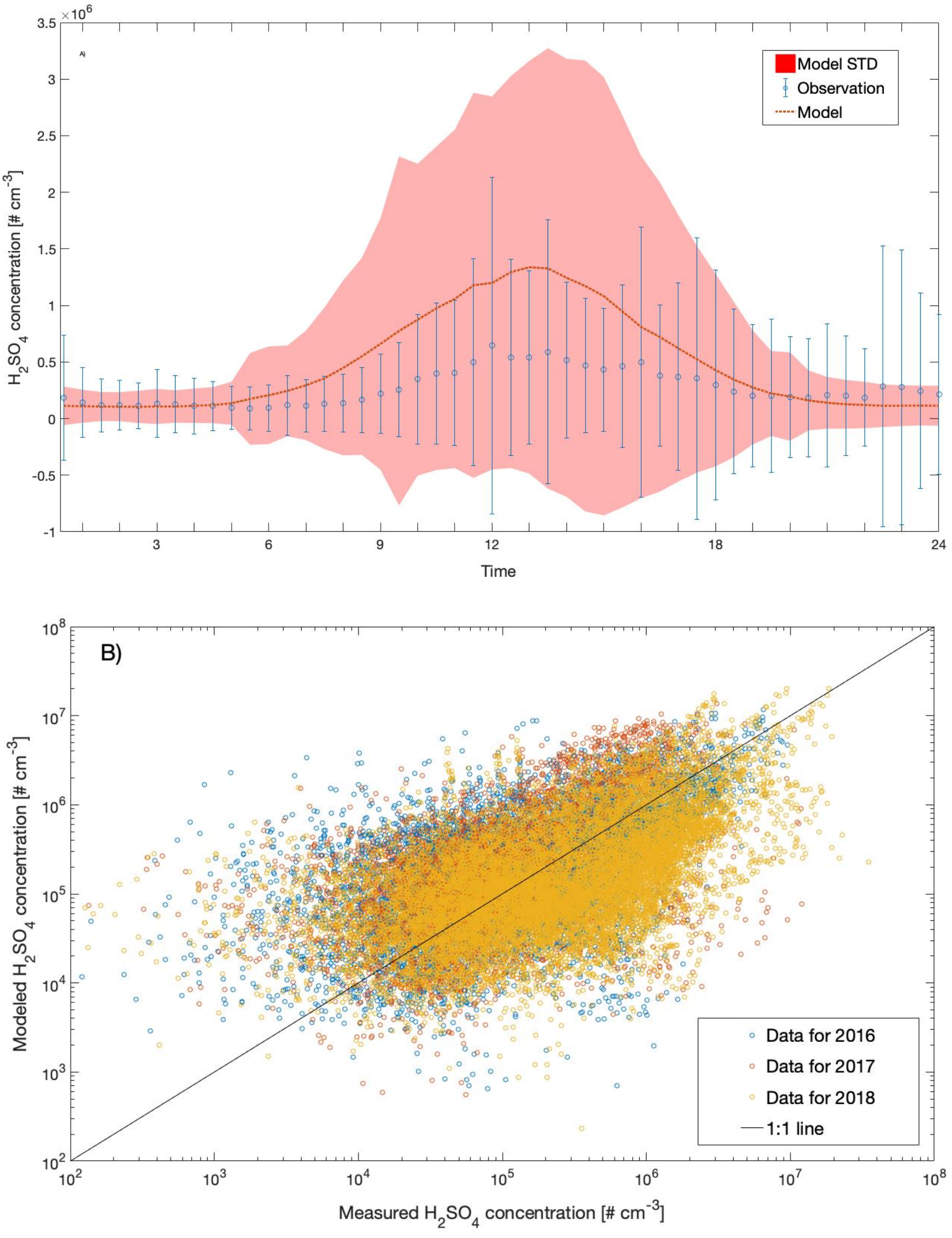

Figure S9: Diurnal cycle of measured and modelled H_2SO_4 concentrations for years 2016-2018 (A); scatter plot for all data points during the 3 years (B).
